# Experimental and theoretical study on the corrosion inhibition of mild steel by nonanedioic acid derivative in hydrochloric acid solution

**DOI:** 10.1038/s41598-022-08146-8

**Published:** 2022-03-18

**Authors:** Ahmed A. Al-Amiery, Abu Bakar Mohamad, Abdul Amir H. Kadhum, Lina M. Shaker, Wan Nor Roslam Wan Isahak, Mohd S. Takriff

**Affiliations:** 1 Renewable Energies and Technology Energy Center, University of Technology-Iraq, Baghdad, 10001 Iraq; 2 Department of Chemical and Process Engineering, Faculty of Engineering and Built Environment, Universiti Kebangsaan Malaysia, 43600 Bangi, Selangor Malaysia; 3 University of Al-Ameed, Karbala, Iraq; 4 Chemical and Water Desalination Engineering Program, Department of Mechanical and Nuclear Engineering, Collage of Engineering, University of Sharjah, 26666 Sharjah, United Arab Emirates

**Keywords:** Chemistry, Materials science

## Abstract

The corrosion performance of mild steel (MS) in 1M HCl solution was examined by weight loss (WL), potentiodynamic polarization (PDP), electrochemical impedance spectroscopy (EIS), electrochemical frequency modulation (EFM), and open circuit potential (OCP) measurements in the absence and presence of nonanedihydrazide. PDP measurements indicated that nonanedihydrazide acts as a mixed inhibitor due to its adsorption on the MS surface, exhibiting an inhibition efficiency of more than 97%. The surface morphology investigation of the protective layer on the MS surface confirmed that adsorption of nonanedihydrazide molecules occurred via chemical adsorption following Langmuir’s isotherm model. The effect of temperature on the corrosion performance in the presence of nonanedihydrazide was investigated in the range of 303–333 K, showing that the inhibition efficiency increased with an increase in the inhibitor concentration and decreased with an increase in temperature. A new green corrosion inhibitor was synthesised and theoretical computations were conducted to completely understand the inhibition mechanism. Nonanedihydrazide molecules were investigated by DFT (density functional theory) using the B3LYP functional to evaluate the relationship of corrosion inhibition performance and the molecular structure. The computed theoretical parameters presented significant support for understanding the inhibitive mechanism revealed by the inhibitory molecules and are in good agreement with WL, PDP, EIS, (EFM), and OCP results.

## Introduction

Corrosion inhibitors are chemicals that efficiently slow the rate of corrosion of metals and alloys when used in relatively low concentrations, particularly in cooling systems, storage vessels and boilers, oil and gas pipelines, as well as in construction. Mild steel (MS) is an important construction material due to its outstanding mechanical characteristics and minimal costs in comparison to other materials^1^. However, MS like other alloys is susceptible to corrosion, thus the surface must be protected^2^. However, this operation needs to be controlled due to the extremely damaging corrosive effect of acids used such as hydrochloric acid. The acidising process in general manufacturing cleaning techniques in petrochemical applications eliminates metal oxides and inorganic layer removals^3^, thus MS corrosion is unavoidable but can be controlled^4^. Incorporating passive fillers into organic coatings to enhance protection against corrosion is one technique to overcome this problem^5,6^. Natural and synthetic organic inhibitors are essential additives for MS corrosion protection^7^, with environmentally friendly natural and/or synthesised organic inhibitor usage developed to meet environmental requirements. Hence, inorganic inhibitors including chromate salts, molybdenum oxoanion, orthophosphate ions, and nitrates are widely utilised as corrosion inhibitors of metallic surfaces (e.g., MS), which are replaced despite their considerable efficacy^8^. Although the organic corrosion inhibitors have environmentally friendly and biodegradable characteristics, they also have corrosion inhibiting properties^9^. Organic corrosion inhibitors have electron donor atoms including phosphorous, sulphur, oxygen and nitrogen, which allow them to be adsorbed on the metal surface and protect the surface from acidic solutions^10^. The corrosion inhibition efficacy displayed by these heteroatoms increases in the order: P > S > N > O^11^. There are various studies on corrosion inhibition by aliphatic amines and hydrazides for various alloys^12^, with amines and hydrazides of fatty acids being more efficient than cyclic amines, hydrazides and aromatic amines^13^. In general, the chemical bonds between metals and natural and/or synthesised organic inhibitor molecules can influence the quality of protective layers and perhaps even corrosion inhibition. Consequently, additional active sites in the inhibitor molecular structure, such as heterocyclic portions, may provide a stronger chelation interaction with steel, allowing for the fabrication of a stronger inhibitor adsorption barrier on the surface^14,15^. DFT is a useful technique to explain experimental findings, allowing and obtaining dependable structural molecular factors^16^. In corrosion investigations, this technique makes it feasible to correctly predict the inhibitive efficacy of natural or synthetic organic molecules based on electronic characteristics in addition to reactivity indices^17^. Nitrogen-rich materials are attractive corrosion inhibitors, with synthetic chemicals accounting for a significant portion of nitrogen-rich corrosion inhibitors^18^. However, natural chemicals derived from plants have recently been demonstrated to possess some very impressive qualities^19^. Plant extracts are also less expensive, easier to obtain, renewable, highly biodegradable, readily available, and particularly non-toxic to the environment^20^. Organic corrosion inhibitors are used in a variety of industries but there have several disadvantages, for example, limited solubility making their use difficult especially in polar electrolytes. Thiosemicarbazide is the most important class of nitrogen-rich organic chemicals produced from thiosemicarbazide^21^. Organic corrosion inhibitors, especially those with aromatic rings and non-polar hydrocarbon chains, have limited solubility due to their hydrophobic nature, thus reducing their protective efficacy. Consequently, contemporary corrosion science and engineering research efforts focus on developing corrosion inhibitors with polar, hydrophilic functional alternatives in their molecular structures. The size, function, and geometry of these molecules are critical parameters that explain the efficacy of corrosion inhibitors. The best corrosion inhibitor has a polar hydrophilic function and non-polar hydrophobic hydrocarbon chain in addition to toxicity and cost. The targeted inhibitor is formulated with natural products and a nitrogen-rich complex. In the current study, a corrosion inhibitor was synthesised and the chemical structure was elucidated based on spectroscopical techniques. It was then investigated as a green and inexpensive efficient inhibitor of MS corrosion in an HCl environment at various temperatures and the thermodynamic and kinetic parameters were also examined. The mechanism of adsorption and inhibitory activity of nonanedihydrazide molecules was measured as an inhibitor employing DFT at the level B3LYP.

## Experimental section

### Materials and methods

The MS samples were purchased from the Company of Metal Samples and utilised as the base substrates for the gravimetric measurements and as working electrodes for the electrochemical corrosion experiments: Fe-99.210%; C-0.210%; Si-0.380%; P-0.090%; S-0.005%; Mn, 0.050%, and Al-0.010%. Each sample had an effective surface area of 4.5 cm^2^ and before every test, silicon carbide sheets (120, 600 and 1200) were used to abrade the MS samples before washing with distilled water, acetone and cleaned based on the standard technique ASTM/G1-03^22^. The 1M HCl assay solution was prepared using 37% analytical grade HCl and double-distilled water. The corrosion measurements were conducted in non-stirring conditions and open to the air acidic media with various nonanedihydrazide inhibitor concentrations.

### Synthesis of the inhibitor

**Figure 1:**
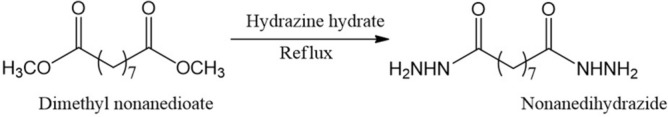
Schematic route for the synthesis of nonanedihydrazide.

Initially, hydrazine hydrate (0.002 mmol) was added to a solution of dimethyl nonanedioate (0.001 mol) and refluxed for 6 h before the refluxed solution was concentrated, filtered, and recrystallised using ethyl alcohol. The inhibitor was purified by TLC and the synthesis is shown in Fig. 1. The nuclear magnetic resonance spectra were recorded using an AVANCE III 600 MHz spectrometer (Bruker, Billerica, MA, USA), FTIR spectra by the Thermo Scientific Nicolate 6700 FT-IR Spectrometer (Thermo Fisher Scientific, Waltham, MA, USA), and the molecule fragments were determined by a GC-FID and GC–MS 7890 A system provided by Agilent Technology (ESI is the source type, Ion Polarity Positive, Set Capillary 4500 V, Set Dry Heater 180 °C, Set End Plate Offset -500 V, Set Dry Gas 5.0 l/min, Scan Begin 100 m/z, Scan End 1000 m/z, Set Collision Cell RF 250.0 Vpp). FT-IR (cm-1): 3313.80, 3289.50, 3199.60 and 3145.00 (NH2-NH-), 3047.00 (Alpha-methylene groups), 2922.40 (Beta-methylene groups), 2849.00 (Aliphatic methylene groups) and 1632.20 cm (carbonyl). Proton-HNMR (CDCl_3_): δ 1.2070 (2H, m, methylene) and δ 1.4120 (2H, m, methylene), δ 1.938.0 (2H, m, methylene) and δ 8.8820 (2H, s, N–H). Carbon-^13^NMR (CDCl_3_): δ 172.140 (carbonyl); 39.640, 39.500, 34.130, 33.860, 33.600, 29.540, and 28.980 (methylene). m/z: [217.160 (C_9_H_20_N4O_2_), 186.140 (C_9_H_18_N_2_O_2_), 156.120 (C_9_H_16_O_2_).

### Gravimetric measurements

Weight loss measurements were conducted based on the standard techniques^22^. The abraded MS samples were weighed before exposure to a 1.0 M HCl environment in the presence and absence of various inhibitor concentrations (0.05, 0.1, 0.2, 0.4, and 0.5 mM). All the acidic environments were aerated and after immersion (1, 5, 10, 24, and 48 h), the samples were removed, washed, dried, and weighed. The tests were performed in triplicate and the average values were calculated. The experiments were repeated with various inhibitor concentrations, temperatures ranging from 303 to 333 K and the immersion time of 5 h. The corrosion rate (C_R_), inhibition efficiency%) and surface coverage (θ) were determined according to Eqs. (1–3):

1 $$C_{R} = \frac{87.6W}{{atd}}$$

2 $$IE\% = \frac{{w_{o} - w_{i} }}{{w_{o} }} \times 100$$

3 $$\theta = 1 - \frac{{w_{i} }}{{w_{o} }}$$

where *w* refers to the MS mass loss (mg), *a* is the MS coupon area (cm^2^), *t* is exposure period (h), *d* is the MS coupon density (g/cm^3^), w describes MS specimen weight loss, w_i_, represents MS coupon mass loss with various concentrations of inhibitor.

### Electrochemical measurements

A Gamry water-jacketed glass cell was used to perform electrochemical studies at a steady-state corrosion rate. MS samples, a graphite rod, and a saturated calomel electrode (SCE) comprised the three-electrode cell setup, which includes working, counter, and reference electrodes. A Gamry Device Potentiostat/Galvanostat/ZRA (REF 600) model was used to conduct electrochemical corrosion analysis (Gamry, Warminster, PA, USA) and the Gamry software was utilised to achieve the corrosion potential, EFM, PDP and EIS measurements. The PDP curves were conducted by adjusting the potential between − 0.2 to + 0.2 VSCE over the corrosion potential at a scan rate of 0.5 mV·s^−1^. EIS measurements were conducted via alternating current signals of 5 mV peak-to-peak amplitude at the corrosion potential using the frequency region of 100 kHz to 0.1 Hz. All impedance data were fitted and simulated to appropriate equivalent circuits (ECs) utilising the Gamry Echem Analyst software. EFM tests were accomplished at a 0.1 Hz base frequency with an alternating current amplitude for 20 cycles. The electrochemical corrosion tests were conducted for 30 min after the working electrode was exposed in the environment to achieve steady-state conditions. The experiments were performed in triplicate with the average values recorded to ensure that the measurements were repeatable.

### Quantum chemical studies

The ground-state geometry was derived with Gaussian 03, Revision C.01^23^, and the valence and polarisation basis set (6-31G +  + (d,p)) was used to optimise to a local minimum without symmetry constraints^24^. A variation of the DFT approach that combines the Becke three-parameter hybrid (B3)^25^ exchange functional with the Lee–Yang–Parr (LYP)^26^ correlation functional (B3LYP)^27,28^ was utilised to evaluate the optimised geometry, frontier molecular orbitals (HOMO, LUMO) energies, and physical parameters for the molecule in the current investigation. Based on DFT-Koopman's theorem^29^, the ionisation potential (I) is related to the E_HOMO_, whereas electron affinity (A) is related to E_LUMO_. The ionisation potential and electron affinity can be calculated according to Eqs. (4 and 5):

4 $$I = - E_{HOMO}$$

5 $$A = - E_{LOMO}$$

The analysis of the natural bond orbital (NBO)^30^ was conducted to determine the electron density distributions, as electron density represents a significant factor in computing the parameters of chemical reactivity. The electronegativity (χ), hardness (η) and softness (σ) were computed according to Eqs. (6–8):

6 $$\chi = \frac{I + A}{2}$$

7 $$\eta = \frac{I - A}{2}$$

8 $$\sigma = \eta^{ - 1}$$

The transferred electrons number (ΔN) was determined based on the DFT approach^31^ by applying the following Eq. (9):

9 $$\Delta N = \frac{{\chi_{Fe} - \chi_{inh} }}{{2( \eta_{Fe} +  \eta_{inh} )}}$$

where χFe is the iron absolute electronegativity, χinh indicates the absolute electronegativity of the inhibitor molecule, ηFe refers to iron absolute hardness and ηinh represents the inhibitor molecule absolute hardness.

In the current investigation, the χFe theoretical value was 7.0 eV and for ηFe was zero.

## Results and discussion

### Confirmation of the nonanedihydrazide structure

**Figure 2:**
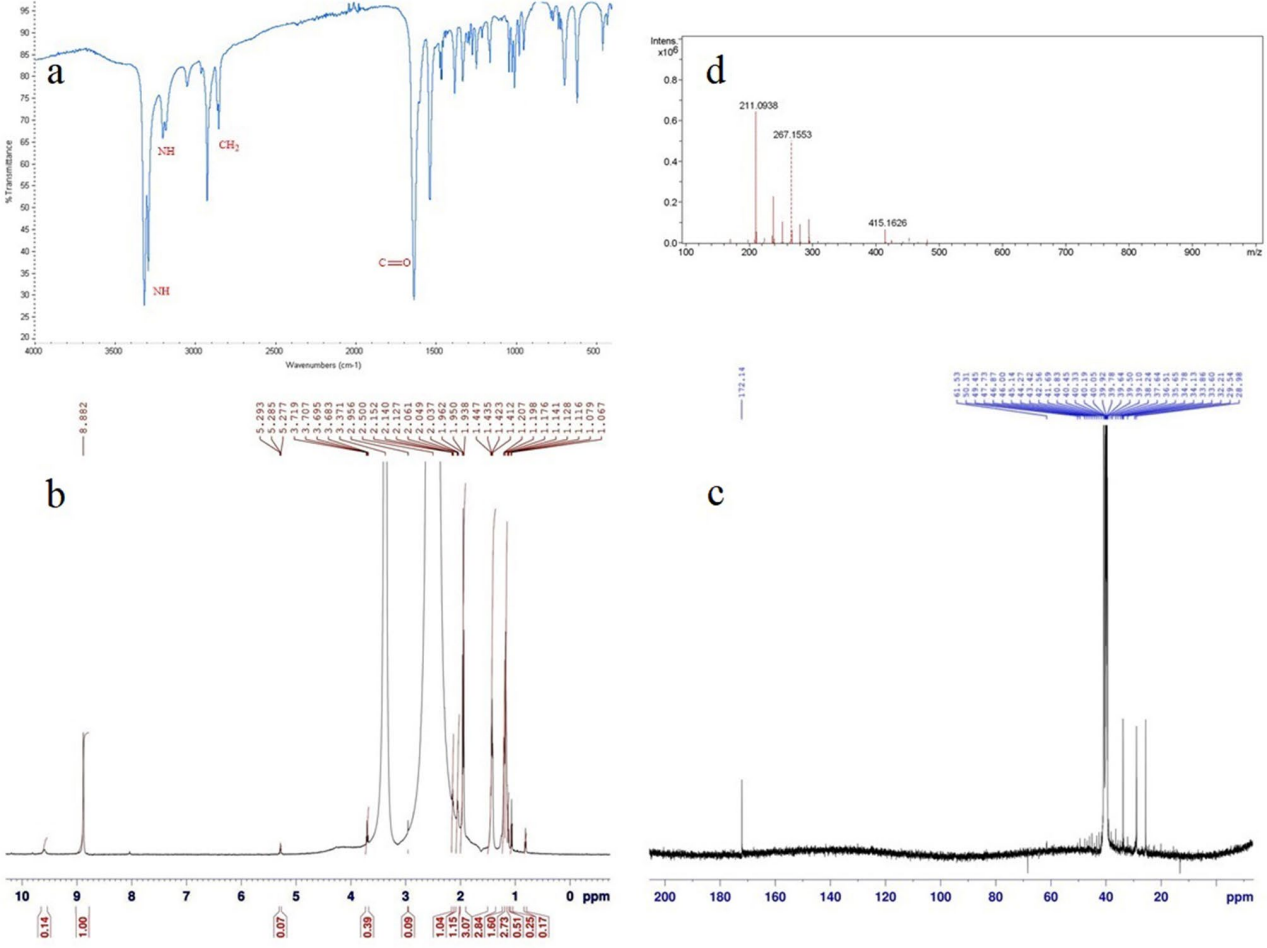
(**a**) FTIR; (**b**) Proton NMR; (**c**) Carbon-^13^ NMR and (**d**) Mass spectra of nonanedihydrazide.

The nonanedihydrazide was prepared via the schematic route shown in Fig. 1 starting with dimethyl nonanedioate. The structure of the synthesised nonanedihydrazide was elucidated via FT-IR, ^1^ H NMR, ^13^C NMR, and mass spectroscopy. The molecular weight of nonanedihydrazide was (216), determined according to the molecular formula (C_9_H_20_N_4_O_2_) and confirmed by mass spectroscopy. Nonanedihydrazide is a corrosion inhibitor dissolved in dichloromethane, acetone, dimethylsulphoxide, dimethylformamide, and alcohol media. Figure 2a shows the FTIR spectrum of nonanedihydrazide, with the band at 3,289 cm^−1^ and 3,314 cm^−1^ corresponding to hydrazide NH_2_ bonds and the band at 3,199 cm^-1^ is a hydrazide NH bond. The significant band at 1633 cm^−1^ is carbonyl (C=O) stretching. Alkyl groups which represent methylene groups (CH_2_) are absorbed in the regions of 2849 cm^-1^ and 2922 cm^-1^.

The ^1^H-NMR spectrum in Fig. 2b shows a singlet at δ 8.882 ppm owing to the protons of an amino group. The multiple singlets for two hydrogens (2H) located at δ 1.207 ppm, δ 1.412 ppm, and δ 1.938 ppm were attributed to the methylene groups.

Figure 2c shows the spectrum of ^13^CNMR, the molecular structure of the synthesised corrosion inhibitor. The band at 172.140 ppm was attributed to the carbon of the C=O group. The carbon atoms of methylene groups also have bands at 28.980 ppm, 29.540 ppm, 33.600 ppm, 33.860 ppm, 34.130 ppm, 39.500 ppm, and 39.640 ppm representing the atoms in the methylene groups. Nonanedihydrazide exhibited an m/z value of 217.0 indicating the carbon–nitrogen cleavage to produce a C=O bond, whereas 186.0 describes the splitting of the nitrogen-carbon linkage yielding C=O, while the m/z value at 156.0 was attributed to a compound with bicarbonyl groups (Fig. 2d).

### Weight loss

#### Effect of the inhibitor concentration

**Figure 3:**
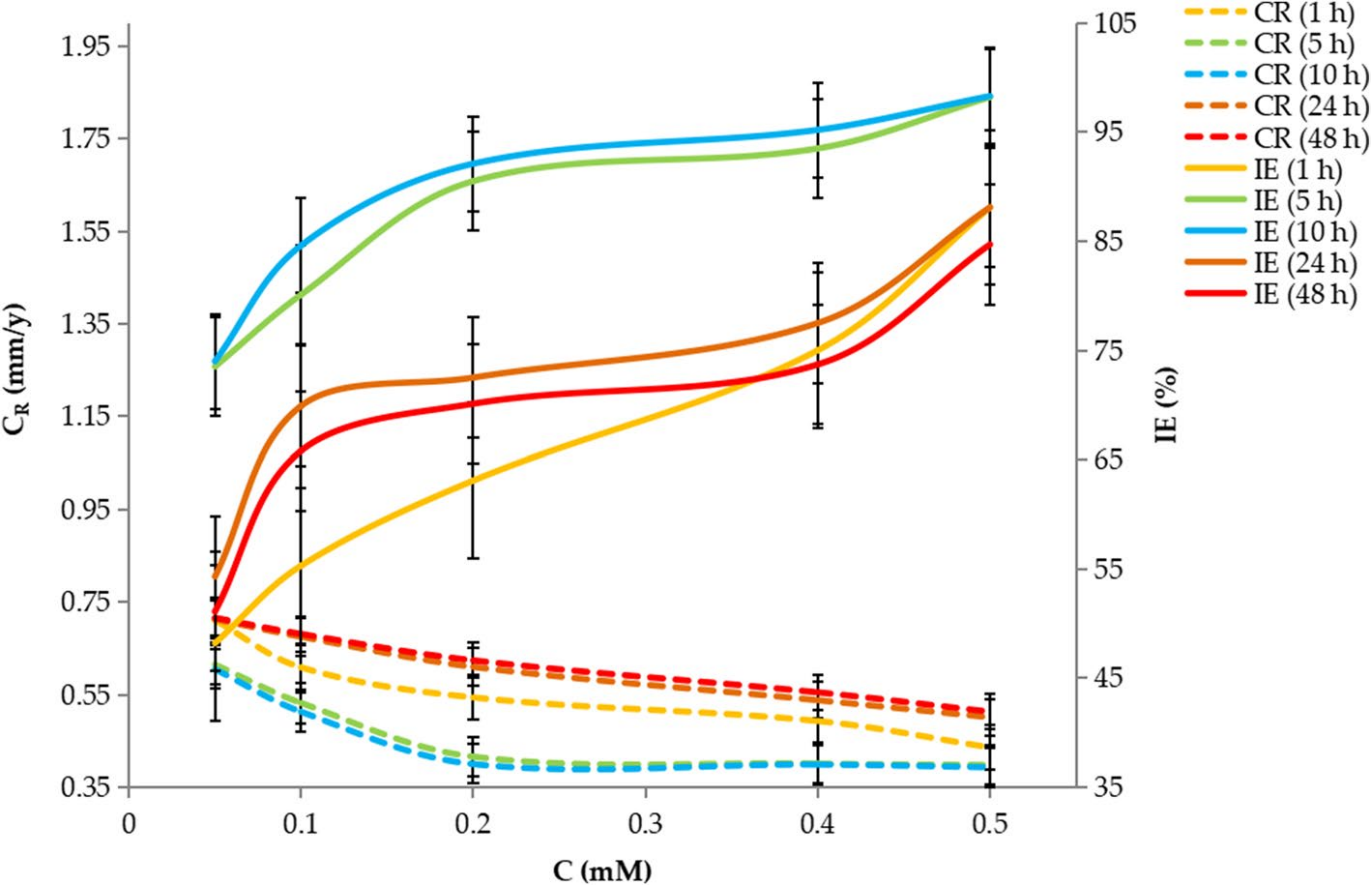
Corrosion rate and inhibition efficiency for MS in 1 M HCl at different immersion times and nonanedihydrazide concentrations.

Nonanedihydrazide protected MS against corrosion and Fig. 3 shows the rate of corrosion C_R_ and inhibition efficiency ($IE\%$). The WL tests were conducted at 303 K, showing that with increasing nonanedihydrazide concentration, the C_R_ decreased, hence, the inhibition improved as more molecules are adsorbed onto the MS decreasing the interaction with HCl. The highest inhibition efficiency (IE)% (98.3%) was exhibited by 0.5 mM nonanedihydrazide and is attributed to the amino and carbonyl groups donating electrons, as well as the inductive effect of methylene groups, which improves the inhibitor’s ability to shift electron pairs to (from inhibitor molecules) the unoccupied d-orbitals of iron atoms on the MS surface, thereby controlling and/or impeding corrosion. Increasing the concentration of nonanedihydrazide to 0.6 mM has no discernible effect on inhibitory performance^32^.

The immersion duration also impacted the resistance of nonanedihydrazide to the HCl environment (Fig. 3), with the C_R_ reducing throughout the first 10 h in 1 M HCl, with the maximum IE of 98.3%, while nonanedihydrazide had the highest IE of 88.1% after 1 h. After 24 h of immersion, the IE value declined, 98.3% at 0.5 mM, reducing to 84.7% after 48 h. The duration of immersion is a critical factor in protection. The adsorption of nonanedihydrazide molecules on the MS surface thereby covering the area of the specimen exposed to the HCl solution may account for the decrease in $C_{R}$ and increase in IE as the concentration of nonanedihydrazide increases^33^. More nonanedihydrazide particles were available to be absorbed onto the MS surface when the concentration of nonanedihydrazide increased, thus the surface coverage significantly increased.

The inhibition efficacy of the synthesised nonanedihydrazide was compared to other corrosion inhibitors having nitrogen atoms to protect against MS corrosion (Table 1). Nonanedihydrazide has the greatest inhibitory efficiency compared to the compounds in Table 1^34–36,38–41^, as well as an efficiency equivalent to that described in^37,42,43^. As the concentration of nonanedihydrazide increased, the rate of corrosion reduced and the inhibitive efficacy improved, possibly because of the increased absorption of nonanedihydrazide on the MS surface as the inhibitor concentration increased.

**Table 1:** Comparison of the inhibitory efficiency of nonanedihydrazide to other triazoles.

Inhibitors	Inhibition efficiencies %	References
Nonanedihydrazide	97	Current inhibitor
Ethyl 2-(4-phenyl-1H-1,2,3-triazol-1-yl) acetate	95.3	^34^
2-(4-phenyl-1H-1,2,3-triazol-1-yl) acetohydrazide	95	^34^
7-((1-benzyl-1H-1,2,3-triazol-4-yl)methyl)-1,3-dimethyl-3,7-dihydro-1H-purine-2,6–dione	91.7	^35^
7-((1-(4-fluorobenzyl)-1H-1,2,3-triazol-4-yl)methyl)-1,3-dimethyl-3,7-dihydro-1H-purine-2,6-dione	86.9	^35^
7-((1-(4-chlorobenzyl)-1H-1,2,3-triazol-4-yl)methyl)-1,3-dimethyl-3,7-dihydro-1H-purine-2,6-dione	94.0	^35^
7-((1-(4-bromobenzyl)-1H-1,2,3-triazol-4-yl)methyl)-1,3-dimethyl-3,7-dihydro-1H-purine-2,6-dione	91.8	^35^
7-((1-(4-iodobenzyl)-1H-1,2,3-triazol-4-yl)methyl)-1,3-dimethyl-3,7-dihydro-1H-purine-2,6-dione	90.9	^35^
5-methyl-4-((3-nitrobenzylidene) amino) -2,4-dihydro- 3H-1,2,4-triazole-3-thione	89.74	^36^
3-phenyl-4-amino-5-mercapto-1,2,4-triazole	97	^37^
2[5-(2-Pyridyl)-1,2,4-triazol-3-yl phenol	96.8	^38^
3,5-Bis(4-methyltiophenyl)-4H-1,2,4-triazole	93.5	^38^
3,5-Bis(4-pyridyl)-4H-1,2,4-triazole	89.1	^38^
3,5-Diphenyl-4H-1,2,4-triazole	82.8	^39^
3,5-Di(*m*-tolyl)-4-amino-1,2,4-triazole	24	^40^
5-Amino-1,2,4-triazole	90	^40^
5-Amino-3-mercapto-1,2,4-triazole	82	^40^
5-Amino-3-methyl thio-1,2,4-triazole	82	^40^
1-Amino-3-methyl thio-1,2,4-triazole	63	^41^
3-Benzylidene amino-1,2,4-triazole phosphonate	56.9	^41^
3-*p*-Nitro-benzylidene amino-1,2,4-triazole phosphonate	69.23	^41^
3-Salicylialidene amino-1,2,4-triazole phosphonate	43.2	^41^
3,5-Bis(methylene octadecyldimethylammonium chloride)-1,2,4-triazole	98.3	^42^
3-Amino-1,2,4-triazole-5-thiol	97.8	^43^

#### Temperature effect

**Figure 4:**
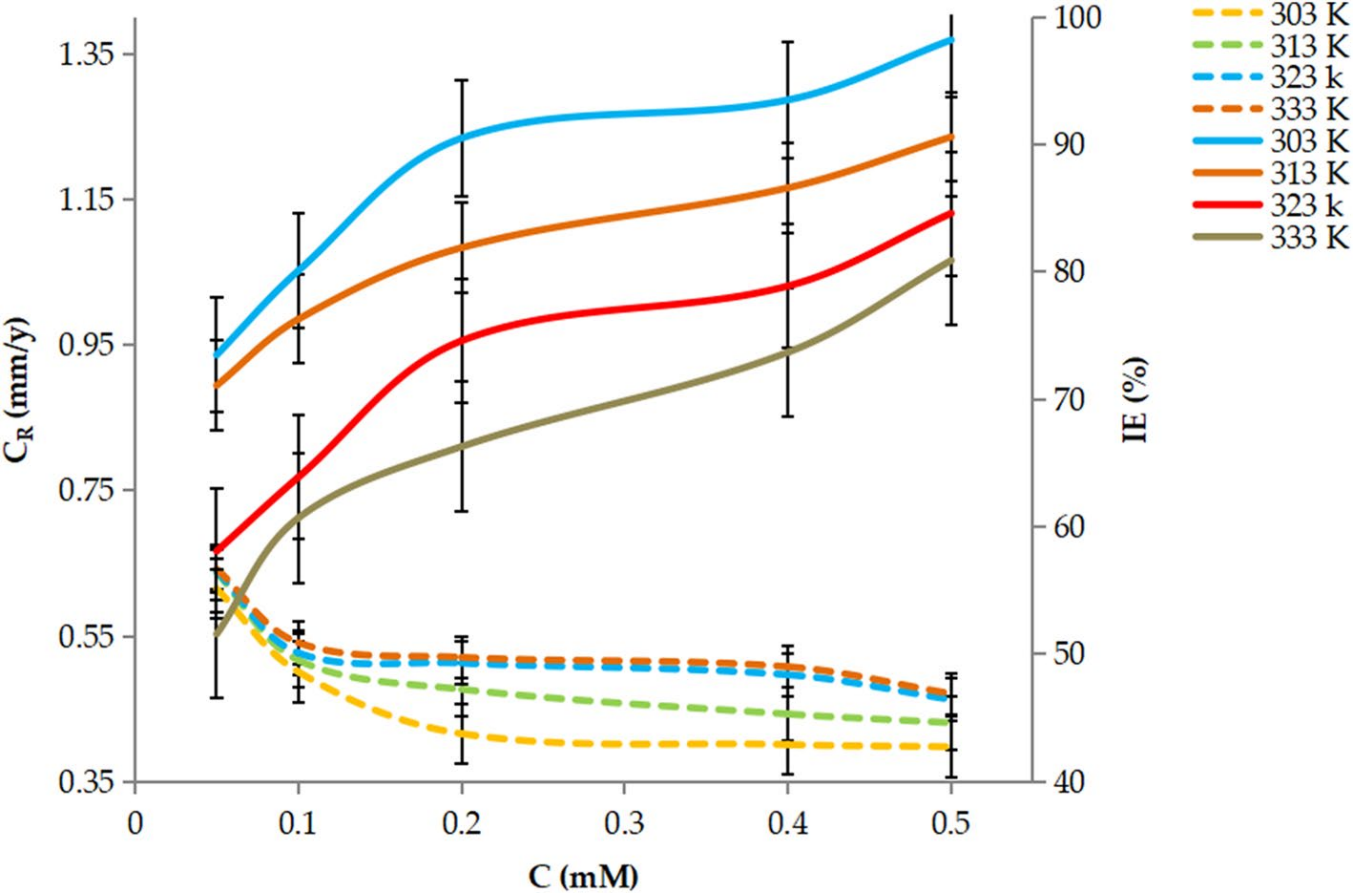
Effect of temperature and concentration on the inhibition efficiency of nonanedihydrazide on MS in 1 M HCl.

Temperature affects the inhibitory efficacy of the inhibitor, with the IE remaining approximately constant as the temperature increased to 323 K, then falls as the temperature climbs to 333 K. Figure 4 shows that at a dose of 0.5 mM, the efficiency of nonanedihydrazide inhibition declines from 98.2 to 73.5% when the temperature changes from 303 to 333 K. This indicates that nonanedihydrazide molecules detach from the MS surface as the temperature rises, so the MS is no longer protected by inhibitor molecules, resulting in a reduction in corrosion IE.

#### Thermodynamic studies

The Arrhenius Eq. (10) and transition state Eq. (11) were used to calculate the temperature-dependent C_R_:

10 $${\text{logC}}_{R} = \frac{{ - E_{a} }}{2.303RT} + \log \lambda$$

11 $$C_{R} = \frac{ - RTT}{{Nh}}\exp \left( {\frac{{\Delta S^{*} }}{R}} \right)\exp \left( { - \frac{{\Delta H^{*} }}{RT}} \right)$$

where E_a_ represents the energy of activation, λ is the factor of pre-exponential and *R* is the constant of gas, $\Delta H^{*}$ is the enthalpy of activation and $\Delta S^{*}$ is the entropy of activation.

**Figure 5:**
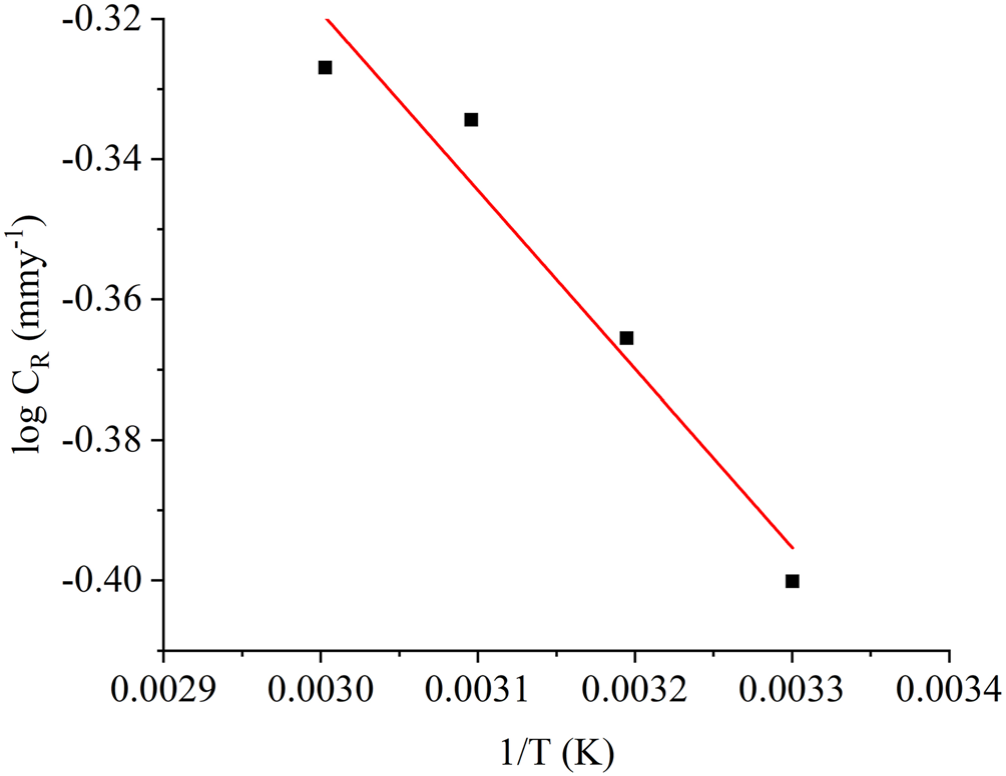
Arrhenius plot $logC_{R} vs\frac{1}{T}$ of nonanedihydrazide optimised concentration (0.5 mM) for 5 h.

The slope of the $logC_{R} vs\frac{1}{T}$ plot (Fig. 5) is −E_a_/2.303R, and the intercept is the value of log λ, thus the pitch and the plotted intercept are used to derive the activation energy Ea and pre-exponential parameter (α). Table 2 shows that in addition to the acid solution, the average activating energies for the inhibited environment were higher. Increasing the activation energy E_a_ a has the same effect as raising the corrosion phenomenon energy barrier, thus improving the IE.

**Table 2:** Thermodynamic parameters for MS corrosion in 1 M HCL with the addition of 0.5 mM nonanedihydrazide.

$E_{a} \left( {{\text{kJ}}\;{\text{mol}}^{ - 1} } \right)$	$\lambda \left( {{\text{mg}}\;{\text{cm}}^{ - 1} } \right)$	$\Delta H^{*} \left( {{\text{kJ}}\;{\text{mol}}^{ - 1} } \right)$	$\Delta S^{*} \left( {{\text{kJ}}\;{\text{mol}}^{{ - {1}}} \;{\text{K}}^{ - 1} } \right)$	$K_{ads} \left( {M \times 10^{4} } \right)$	$- \Delta G_{ads}^{o} \left( {{\text{kJ}}\;{\text{ mol}}^{ - 1} } \right)$
45.7	2.87 × 10^9^	46.9	-77.5	9.6	40.8

As shown in Eq. (11), the C_R_ is dependent on E_a_ and α. To evaluate the MS corrosion inhibition, the activation energy regulates the pre-exponential component, with $E_{a}$ and α values steadily increasing in succession in the current condition. The rate of corrosion is also described in Table 2, indicating that the E_a_ is an initial characteristic for metal corrosion in an HCl environment. An increase or decrease in the free E_a_ differential can indicate favourable physisorption or chemisorption interactions^44,45^. Although the E_a_ for the inhibited environment is higher than for the untreated acidic environment in the current study, the difference is not significant to validate the interactions as physical adsorption or chemical adsorption. As a result, the studied inhibitor compounds and MS surface have physical adsorption and chemical adsorption interactions. Furthermore, from the slope and intercept of the plot of log *C*_R_/*T* versus 1/*T* in Fig. 5, the activation enthalpy $\Delta H^{*}$, and activation entropy $\Delta S^{*}$ were obtained as shown in Table 2. Following the gradual dissolution of MS, the endothermic reaction is denoted by $\Delta S^{*}$ with a (+) value^46^. When the activation entropy values in Table 2 are compared, the activation entropy values increase with the addition of the inhibitor. Eventually, the adsorption preceded the desorption of water molecules from the MS surface, resulting in a rise in $\Delta S^{*}$, which could be attributable to an increase in solution entropy.

#### Adsorption isotherm

Various adsorption isotherm models can be used to get important details based on inhibitor molecules adsorption on the MS surface and as the linear regression coefficient is close to one, the Langmuir isotherm was considered appropriate to describe the inhibitor molecules' adsorption on the MS surface^47,48^. The Langmuir adsorption isotherms can be understood as:

12 $$\frac{{C_{inh} }}{\theta } = \frac{1}{{K_{ads} }} + C_{inh}$$

**Figure 6:**
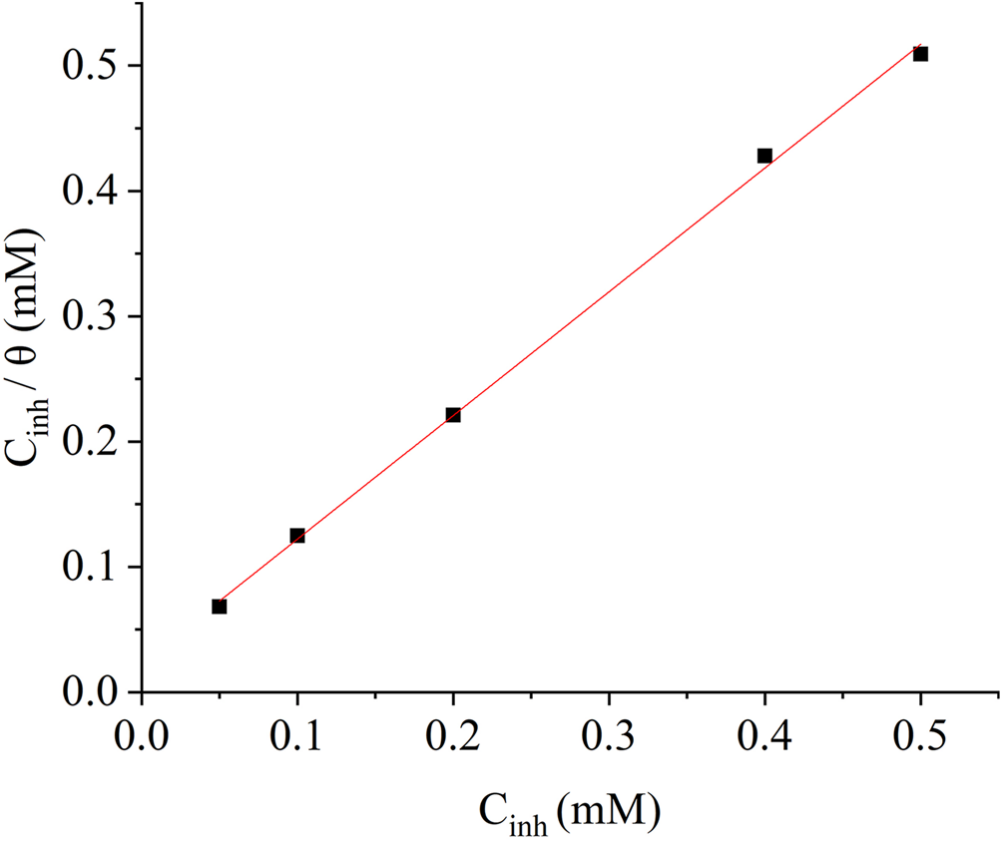
Langmuir adsorption model plot for nonanedihydrazide.

This graph shows the association between the inhibitor concentration C_inh_ and inhibitor surface coverage (θ) on the MS surface. The surface coverage percentage is defined as the proportion of the surface that is coated with inhibitor molecules and can be calculated using Eq. (3). The plot of C_inh_ / θ vs C_inh_ (as shown in Fig. 6) at 303 K is a straight line, indicating that the inhibitor molecules' adsorption temperature is more closely aligned with the Langmuir adsorption temperature than other isotherms. The link between the adsorbent and the adsorbate is represented by K_ads_, with an increased K_ads_ predicting better adsorption, thus better inhibition. The straight-line intercept shown in Table 2 can be used to calculate K_ads_. The link between the adsorption free Gibbs energy and the adsorption equilibrium constant is represented by Eq. (13):

13 $$\Delta G_{ads}^{o} = - RT\ln (55.5K_{ads} )$$

where R refers to the constant of gas, T is the temperature, and K_ads_ is the constant of equilibrium.

The negative value of the adsorption Gibbs free energy $\Delta G_{ads}^{o}$ denotes spontaneity and the inhibitor molecules are adsorbed on the MS^49^. $\Delta G_{ads}^{o} \le - 20\;{\text{kJ}}\;{\text{mol}}^{ - 1}$ represents the physical adsorption of the inhibitor molecule to the MS surface. A substantially negative adsorption free energy $\ge - 40\;{\text{kJ}}\;{\text{mol}}^{ - 1}$ suggests a chemical adsorption reaction and the establishment of coordination interactions between the nonanedihydrazide molecules and the MS surface iron atoms. The value of $\Delta G_{ads}^{o}$ was estimated to be $\ge - 40\;{\text{kJ}}\;{\text{mol}}^{ - 1}$, indicating that the mechanism includes both physical and chemical adsorptions, suggesting mixed-mode interactions^50,51^.

### Electrochemical results

#### Open circuit potential (OCP)

**Figure 7:**
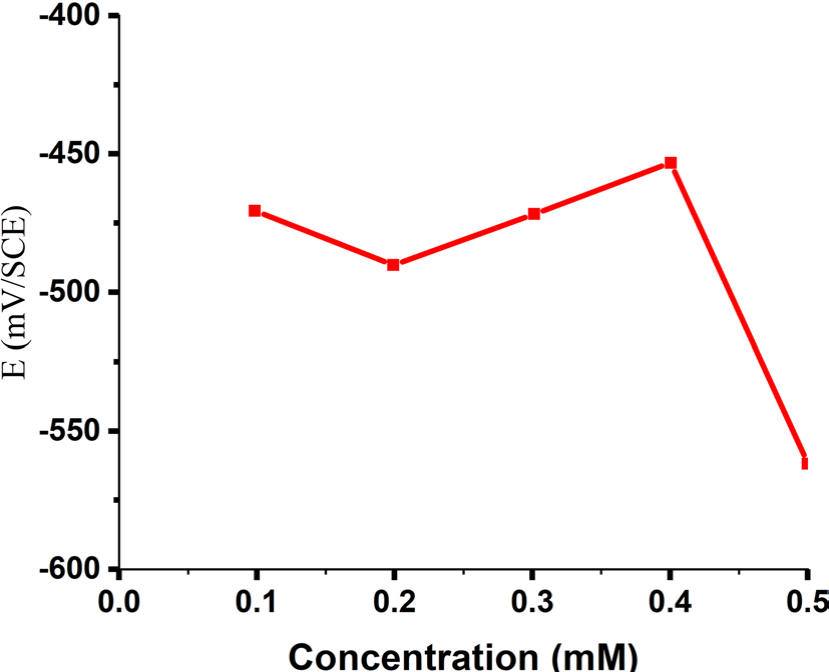
OCP as a function of the nonanedihydrazide concentration for MS in 1.0 M HCl at 303 K.

**Figure 8:**
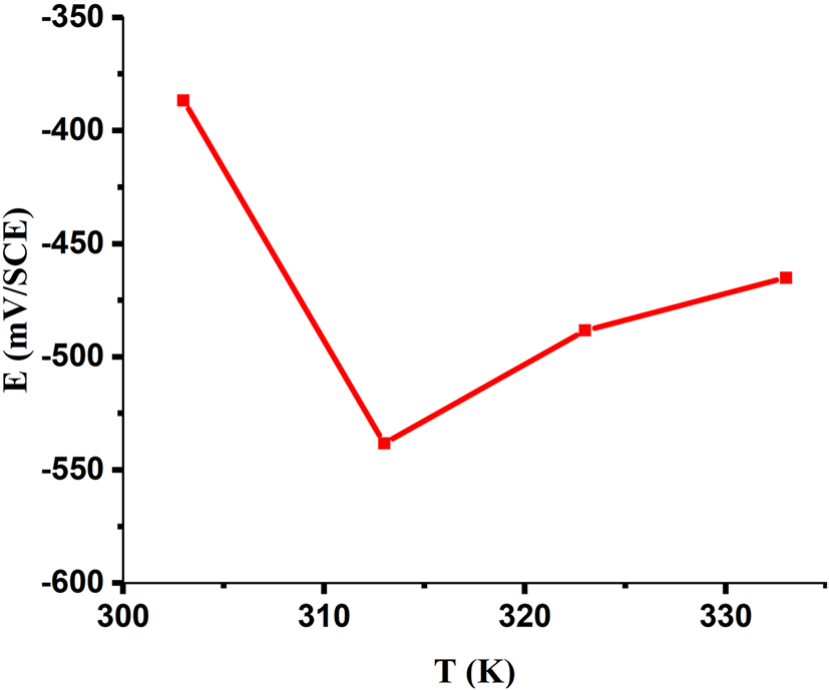
OCP as a function of temperature for MS in 1.0 M HCl and 0.5 mM nonanedihydrazide.

The *open-circuit potential* for MS in 1.0 M HCl at 303 K is shown in Fig. 7 as a function of the nonanedihydrazide concentration. The MS sample is the negatively charged electrode, while nonanedihydrazide is the positively charged solution^52,53^. Adsorption to the metal surface, which is usually made up of free oxides, is a fundamental stage in acidic solution inhibition^54^. Variations in the OCP of 90.70 mV vary from −560.90 to −470.20 mV, implying that nonanedihydrazide molecules are absorbed through a positively charged protecting film on the surface of the negatively charged MS specimens^55^. The OCP dramatically decreases between 303 and 333 K at the same concentration of 0.5 mM but at different temperatures due to the temperature difference as in Fig. 8. The OCP rises between 303 and 333 K, indicating that there is corrosion, that is, high temperatures reduce the corrosion inhibitor's effectiveness.

#### EIS

Tables 3 and 4 summarise the EIS measurements for MS corrosion in the presence and absence of the inhibitor at 303, 313, 323, and 333 K. Figure 12 shows the Nyquist plots of the impedance spectra for MS samples in 1.0 M HCl in the absence and presence of various amounts of nonanedihydrazide at 303 K.

**Table 3:** CPE data for MS in 1.0 M HCl with various concentrations of nonanedihydrazide at 303 K.

Conc. (mM)	CPE_dl_		C_dl_ (µF.cm^-2^)	R_ct_ (ohm.cm^2^)	R_s_ (ohm.cm^2^)	IE %
	Y_o_ (µS.s^α^ cm^-2^)	α				
0.0	0.0009	0.9174	3.387	0.0781	0.2537	0.00
0.05	0.0051	0.7096	1.375	0.7617	0.3595	89.75
0.1	0.0038	0.7258	0.8209	0.7754	0.3567	89.93
0.4	0.0017	0.8006	0.3659	0.7856	0.3927	90.06
0.5	0.0004	0.8584	0.2662	0.8479	0.5489	90.78

**Table 4:** CPE data for MS in 1.0 M HCl with nonanedihydrazide (0.5 mM) at various temperatures.

Temp**. (**K)	CPE_dl_		C_dl_ (µF.cm^-2^)	R_ct_ (ohm.cm^2^)	R_s_ (ohm.cm^2^)	IE %
	Y_o_ (µS s^α^ cm^-2^)	α				
303						
Without Inh	924.6	0.9174	338.7	0.0781	0.2537	0.00
With 0.5 mM	435.4	0.8584	266.2	0.8479	0.5489	90.78
313						
Without Inh	4526	0.9278	502.0	0.2195	0.2478	0.00
With 0.5 mM	500.2	0.8381	398.5	0.3521	0.2311	73.66
323						
Without Inh	1634.01	0.7321	835.9	0.1501	0.2305	0.00
With 0.5 mM	507.5	0.8392	266.1	0.3378	0.2077	55.57
333						
Without Inh	2172.87	0.8470	920.8	0.1193	0.1836	0.00
With 0.5 mM	451.4	0.8619	554.5	0.2490	0.1756	52.09

**Figure 9:**
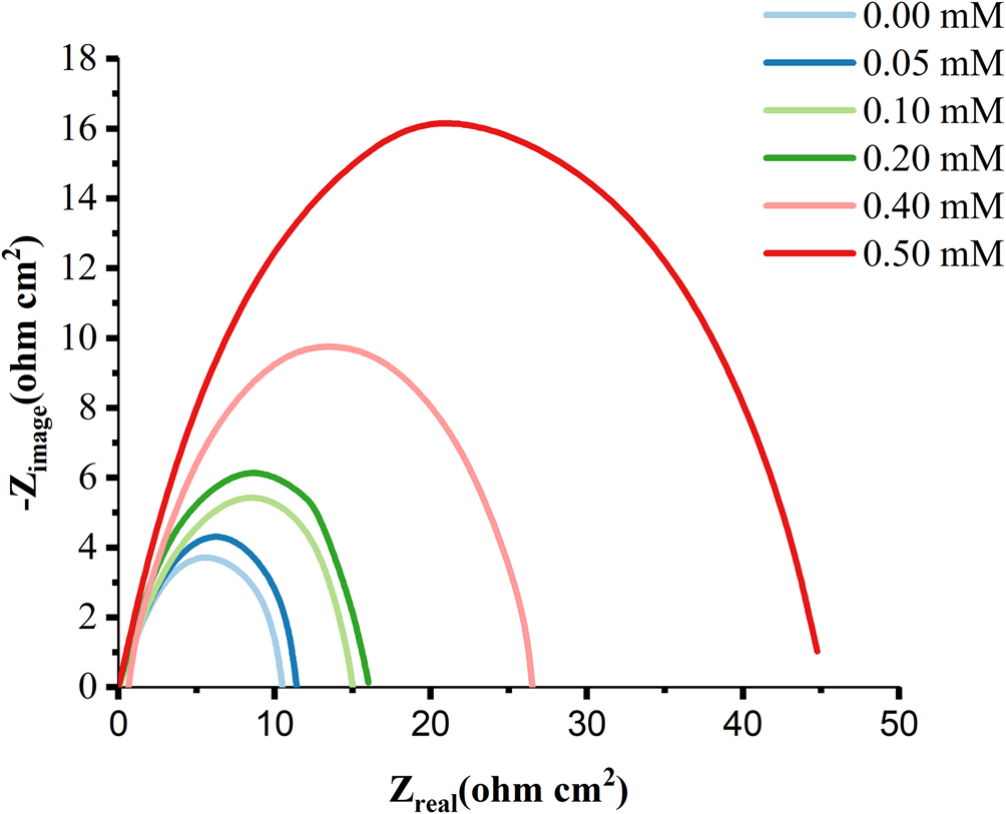
Nyquist plots for MS in 1.0 M HCl with various concentrations of nonanedihydrazide at 303 K.

**Figure 10:**
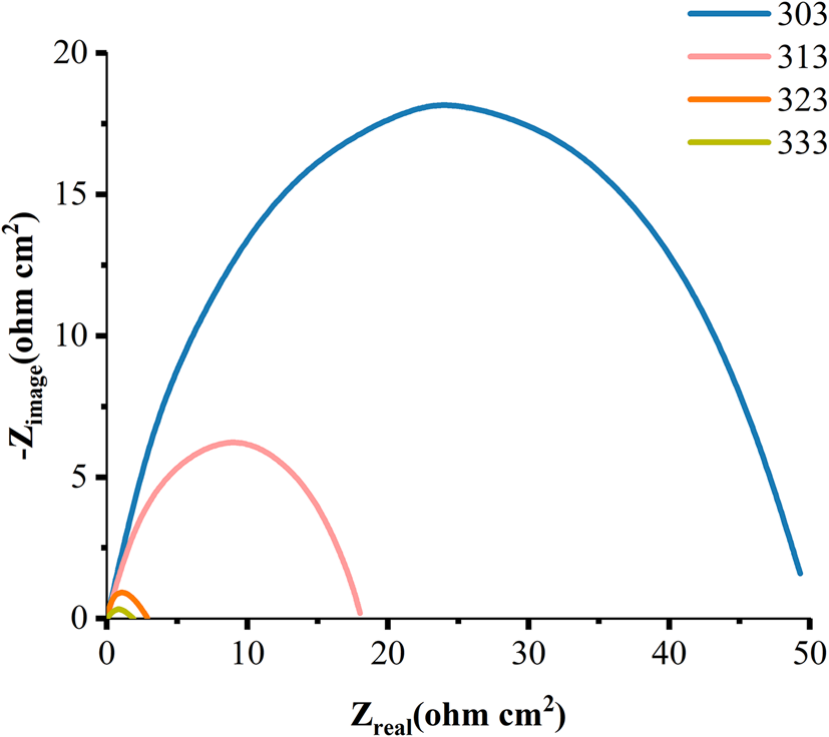
Nyquist plots for MS in 1.0 M HCl with 0.5 mM nonanedihydrazide at various temperatures.

**Figure 11:**
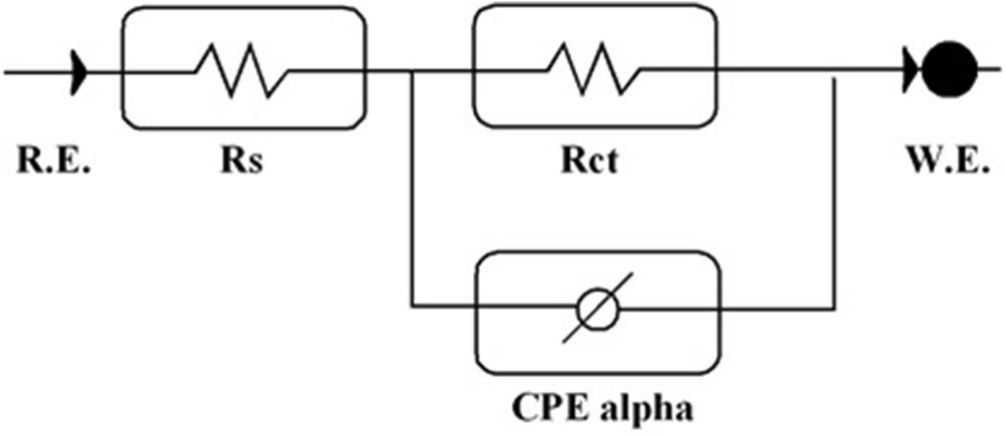
Equivalent circuit model utilised to fit impedance data in 1.0 M HCl with and without the addition of nonanedihydrazide.

The addition of the corrosion inhibitor results in a significant increase in the overall impedance. The impedance response of MS is dramatically affected by the addition of the inhibitor to the corrosive solution, as illustrated in Fig. 9. Increasing inhibitor concentration causes an increase in substrate impedance and the total impedance of MS in the presence of 0.5 mM decreases as the solution temperature rises, as shown in Fig. 10, due to the desorption of adsorbed inhibitor molecules from the MS surface. The Nyquist plots show two loops in the impedance spectrum of MS, namely, in the high-frequency region (HF) and another in the intermediate frequency region (MF), with minimal inductive action at low frequencies (LF). The electrode and charge transfer processes are responsible for the HF and MF loops, respectively. In the absence and presence of the inhibitor, the inductive behaviour seen in the LF region is attributed to the relaxation of the adsorption of corrosion products or the adsorption of inhibitor molecules on the MS surface in an acidic solution^56^. All other temperatures studied show a similar pattern of activity. The working electrode's corroding surface is rough, so the capacitance is given using a constant phase element (CPE). As illustrated in Fig. 11, the EIS findings were examined using the equivalent circuit^57^. Using Eq. (14), the inhibition efficiencies were determined from the charge transfer resistance:

14 $$(IE\% ) = \frac{R(inh) - R(uninh)}{{R(inh)}} \times 100$$

where R_oct_ and R_ct_ indicate the charge transfer resistance in the presence and absence of the corrosion inhibitor, respectively.

The Gamry Analyst software^58^ was utilised to calculate the EIS experimental data, which include data matching CPE for MS/sample calculation solution resistance Rs and CPE, calculation of charge transfers resistance Rct, and double-layer charge, Cdl. Table 3 shows the comparison of the CPE of MS in 1.0 M HCl with various corrosion inhibitor concentrations at 303 K. The Rct value increased as the concentration of corrosion inhibitor increased, indicating that corrosion inhibitor molecules are adsorbed on the surface of MS samples to form a protective layer while the high resistance to charge transfer corresponds to the systems that corrode slowly^59^. At a concentration of 0.5 mM, the efficiency and capacity of inhibition (IE) increased to 90.78% as the value of Rct increased.

Table 4 shows that as the temperature is increased from 303 to 333 K, the Rct also increased, and the inhibition efficacy decreases. Since the corrosion inhibitor molecules are adsorbed on the surface of the MS sample, they will condense as the temperature rises^60^. The physical and chemical adsorptions are the two basic types of adsorption of organic molecules. The presence of a transition metal, the vacuum region, the low energy of the electron orbital, and the inhibitors of molecules with relatively loose electrons are all important factors contributing to corrosion inhibition^61^. Rct values increase with increasing concentration but decrease dramatically with increasing solution temperature, as indicated in Tables 3 and 4. Figure 10 shows the Nyquist plots for MS in 1 M HCl with 0.5 mM nonanedihydrazide at various temperatures. The semicircle graph at 333 K is the shortest while the circle for 303 K is the largest indicating that when the temperature increases the diameter of the semicircle shrinks. These results indicate that as the temperature increases, the corrosion prevention decreases significantly and the corrosion accelerates with increasing temperature due to changes in the corrosion actuation mechanism^55^.

**Figure 12:**
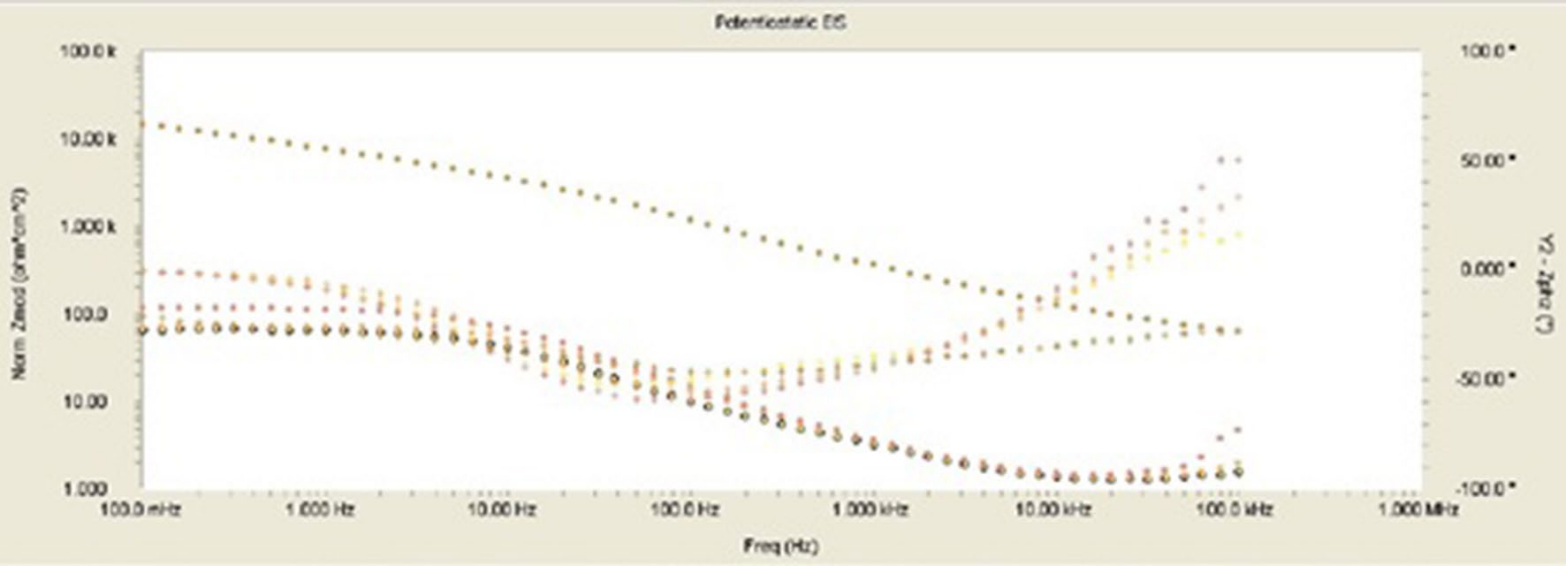
Experimental impedance and phase data in Bode format for MS in 1.0 M HCl containing 0.5 mM nonanedihydrazide denotes the fitted line using the equivalent circuit.

The inhibitor-covered metal/solution interface is described using the equivalent circuit model depicted in Fig. 11, while Fig. 12 shows a one-time constant in the Bode phase. Figure 12 depicts the impedance and phase data in the form of Bode graphs for MS exposed to 1 M HCl at 303 K and shows the fit of the line using the parabolic circuit model.

Figure 12 shows Bode diagrams for MS in 1 M HCl with and without the corrosion inhibitor. In the EIS spectrum, different wear regimes (e.g., charge transfer control, diffusion control, or mixed-type) may reveal different features. The process of system erosion can be identified by studying the EIS data (Nyquist plot, Bode plot). EIS data are often interpreted in terms of equivalent electrical circuits that can be used to characterise the electrical properties of electrochemical communications in practice^56^. Most impedance spectra reported for MS corrosion in hydrochloric acid solutions include one low capacitive loop (one time constant in the Bode phase representation) or two capacitive semicircles (two well-defined time constants in the Bode phase format).

When the Nyquist plot contains a 'low' semicircle with the centre below the real axis, this is known as frequency dispersal and has been linked to surface roughness and inhomogeneity^57^. EIS spectra of heterogeneous coatings on metallic surfaces or rough and porous electrodes have been described by two approaches, namely the finite transmission line model^58^ and the illustrated equivalent circuit model, both of which are generally recommended in the analysis of the degradation of coated metals. The EIS spectra of the metal covered with films of organic inhibitors were compared with the spectra of the failed coating metals^59^.

#### Polarisation measurements

**Figure 13:**
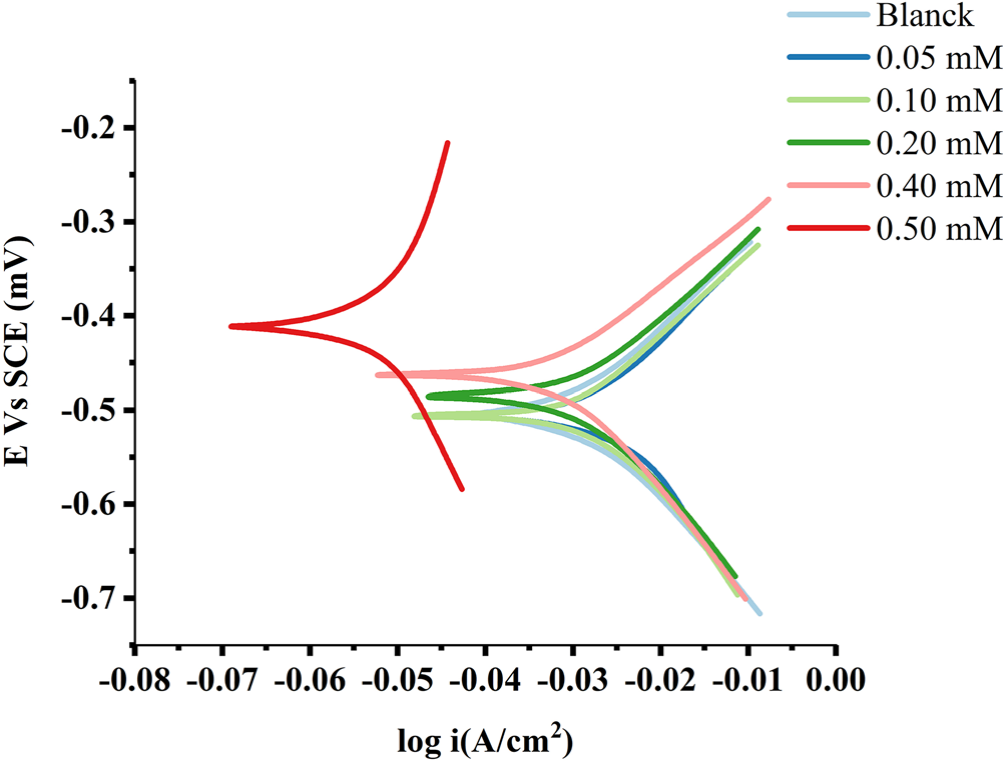
Potentiodynamic polarization curves for MS in 1.0 M HCl with different concentrations of nonanedihydrazide at 303 K.

**Figure 14:**
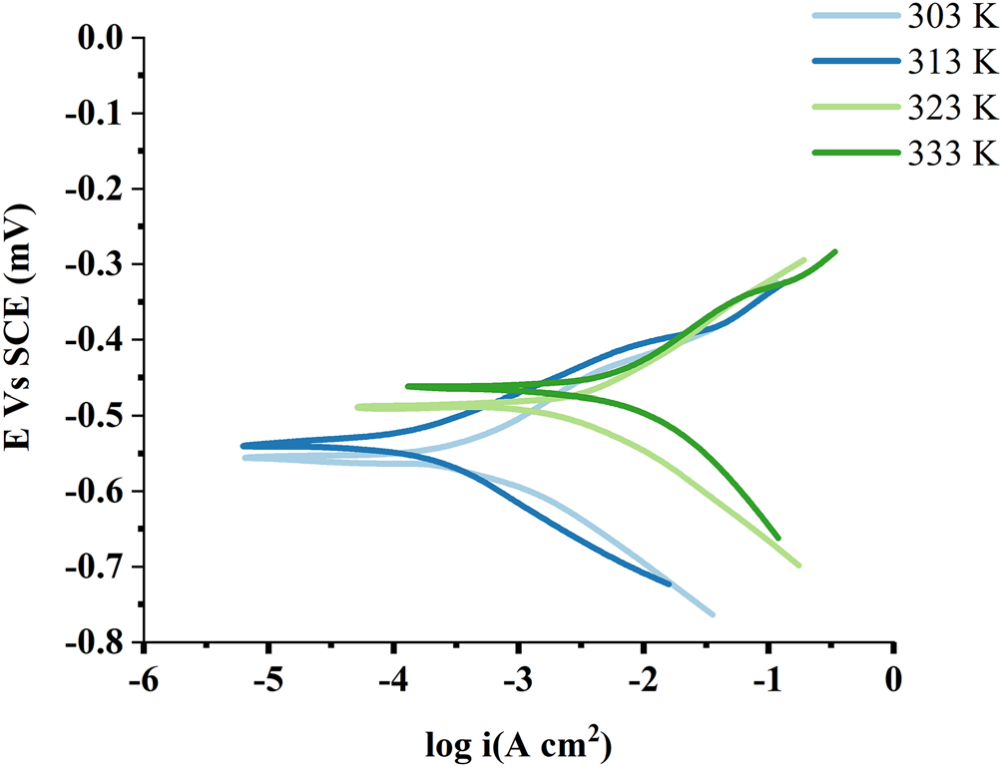
Potentiodynamic polarization curves for MS in 1.0 M HCl with 0.5 mM nonanedihydrazide at various temperatures.

Figures 13 and 14 show the polarisation profile of MS in 1.0 M HCl. Tables 5 and 6 show the numerical values of fluctuations of erosion current density (i_corr_), corrosion potential (E_CORR_), anodic Tafel slope (a) and cathodic Tafel slope (c) with different nonanedihydrazide concentrations and at different solution temperatures for a specific polarisation profile. The intersection of the anodic and cathodic Tafel lines of the polarisation curve at ECORR yielded these results and Eq. (15) was used to compute the IE:

15 $$(IE\% ) = \frac{{i_{corr}^{^\circ } - i_{corr} }}{{i_{corr}^{^\circ } }} \times 100$$

**Table 5:** Polarisation parameters for MS in 1.0 M HCl with different nonanedihydrazide concentrations.

Inhibitor conc. (mM)	Potentiodynamic polarization parameters (PD)				
	C_R_ (mpy)	I_corr_ (µA.cm^-2^)	β_c_ (V.dec^-1^)	β_a_ (V.dec^-1^)	IE %
0.0	7.5910	667.00	0.1315	0.1359	0.00
0.05	6.9640	598.00	0.1276	0.1289	10.34
0.1	4.7360	407.00	0.1217	0.1104	38.98
0.2	3.9950	343.00	0.1184	0.1012	48.58
0.4	2.0580	177.00	0.1138	0.0877	73.46
0.5	0.0537	44.620	0.4030	0.6004	93.31

**Table 6:** Polarisation parameters for MS in 1.0 M HCl with 0.5 mM nonanedihydrazide at variable temperature conditions.

T. (K)	Potentiodynamic polarization parameters (PD)					
	C_R_ (mpy)	I_corr_ (µA.cm^-2^)	β_c_ (V.dec^-1^)	β_a_ (V.dec^-1^)	−E_corr_ (mV.vs.SCE)	IE %
303						
Without Inh	7.5910	550.000	0.13150	0.13590	493.000	0.000
With 0.5 mM	0.0537	44.6200	0.40300	0.60040	385.000	93.31
313						
Without Inh	76.6600	667.000	0.67750	0.70330	655.000	0.000
With 0.5 mM	13.6900	135.000	0.09630	0.07070	538.000	75.45
323						
Without Inh	114.800	824.000	0.71790	0.97220	674.000	0.000
With 0.5 mM	423.100	416.000	0.12900	0.12010	487.000	49.51
333						
Without Inh	622.400	1564.000	2.00300	4.01500	673.000	0.000
With 0.5 mM	1017.00	999.000	0.14240	0.14240	463.000	36.13

where $i_{corr}^{o}$ and $i_{corr}$ are the corrosion current densities in the absence and presence of the inhibitor, respectively.

Tables 5 and 6 show that as i_corr_ increases with increasing solution temperature while decreasing with the addition of the synthetic inhibitor to the acidic solution over the studied temperature range. This result can be described as follows: the inhibitor is adsorbed on the metal surface, and as the temperature rises, some of the inhibitor molecules adsorb the adsorbent, exposing more of the metal surface to the acidic medium, which increases the rate of metal dissolution and decreases the inhibition efficiency^60^. The addition of the inhibitor induces the selected ECORR values to adjust towards more positive values, indicating that nonanedihydrazide has an inhibitory effect on the corrosion of MS at 303 K. Nevertheless, this value decreases with the temperature of the solution, indicating a low level of nonanedihydrazide protection. The anodic and cathodic processes are changed accordingly when different amounts of nonanedihydrazide are added, as indicated in Figs. 13 and 14. When the change in E_CORR_ is greater than 85 mV, the tested inhibitor is classified as an anodic or cathodic type inhibitor^61^. Nonanedihydrazide acts as a mixed-type inhibitor as its highest displacement is 385 mV at 303 K (Table 5), indicating that the addition of an acidic solution reduces the anodic solubility of MS and delays the formation of cathodic hydrogen.

#### Electrochemical frequency modulation (EFM)

EFM is an electrochemical approach for estimating the corrosion rate without knowing the Tafel constants in advance^62^. A major advantage of this method is that it measures the erosion rate, Tafel parameters, and causation variables in one data set. To produce a current response using EFM, a potential perturbation signal consisting of two sine waves is applied to any corrosion sample. Table 7 shows that as the inhibitor concentration rises, the i_corr_ rate decreases, CF-2 and CF-3 have standard values of 2.0 and 3.0, respectively. If the value of the causation factor approaches the criteria, there is a correlation between the perturbation and response signals, and the data can be accepted. Electrochemical frequency modulation has been used to accurately evaluate corrosion parameters for a variety of metals and electrolytes. This method is similar to the harmonic method in that it uses a low amplitude (20 mV) sine perturbation signal but instead of one sine wave, it uses two sine waves. Data validation, larger current response, insensitivity to harmonics and perturbation signal are just some of the benefits of electrochemical frequency modulation over the harmonic technique. Tables 7 and 8 show the corrosion parameters for different concentrations of nonanedihydrazide in 1.0 M HCl at 303 K and different temperatures, respectively for protection efficacy, corrosion current density (A•cm^2^), Tafel constant, and causal factors (CF-2) and (CF-3).

**Table 7:** Electrochemical frequency modulation (EFM) parameters for MS in 1.0 M HCl with different concentrations of nonanedihydrazide at 303 K.

Conc. (mM)	C_R_ (mpy)	I_corr_ (mA.cm^-2^)	β_2_ (mV.dec^-1^)	β_1_ (mV.dec^-1^)	CF-3	CF-2	IE %
0.0	390.30	3.7590	93.750	81.670	3.2600	1.1010	0.000
0.05	288.30	2.8320	156.50	104.40	2.2410	2.0380	24.66
0.1	166.70	1.6370	124.90	88.050	2.8450	1.9930	56.45
0.2	161.80	1.5900	132.60	89.540	3.4070	1.9360	57.70
0.4	88.860	0.8730	121.80	90.810	5.0510	2.0630	76.78
0.5	31.120	0.0096	111.10	104.30	3.6720	1.6640	99.70

**Table 8:** EFM parameters for MS in 1.0 M HCl with 0.5 mM nonanedihydrazide at various.

Temp. (K)	C_R_ (mpy)	I_corr_ (mA.m^-2^)	β_2_ (mV.dec^-1^)	β_1_ (mV.dec^-1^)	CF-3	CF-2	IE %
303							
Without Inh	390.30	3.75900	93.750	81.670	3.260	1.101	0.00
With 0.5 mM	31.120	0.00960	111.10	104.30	3.672	1.664	99.70
313							
Without Inh	692.80	6.80500	111.90	89.930	2.994	1.994	0.00
With 0.5 mM	169.40	1.6660	107.70	86.370	4.634	2.009	75.52
323							
Without Inh	1948.0	19.140	109.60	93.680	5.767	2.057	0.00
With 0.5 mM	361.10	3.5520	123.30	86.850	3.307	1.948	61.44
333							
Without Inh	5776.0	56.740	146.50	119.60	2.916	1.813	0.00
With 0.5 mM	1104.0	10.850	192.50	144.00	3.135	1.899	60.87

EFM results are valid if CF-2 and CF-3 are in the 0–2 and 0–3 ranges, respectively. Any difference from the theoretical value in the causation factor could be due to a very small perturbation amplitude, poor frequency-resolution of the spectrum, or a defective damper^63^. The inhibition efficacy of nonanedihydrazide increases with the increasing concentration of the inhibitor but decreases with increasing solution temperature at a constant concentration, as previously shown in other tests. This finding demonstrates that the inhibitor molecules are physically rather than chemically adsorbed on the surface of MS and that higher temperature accelerates both the dissolution of the metal and the adsorption of the inhibitor molecule from the metal surface.

**Figure 15:**
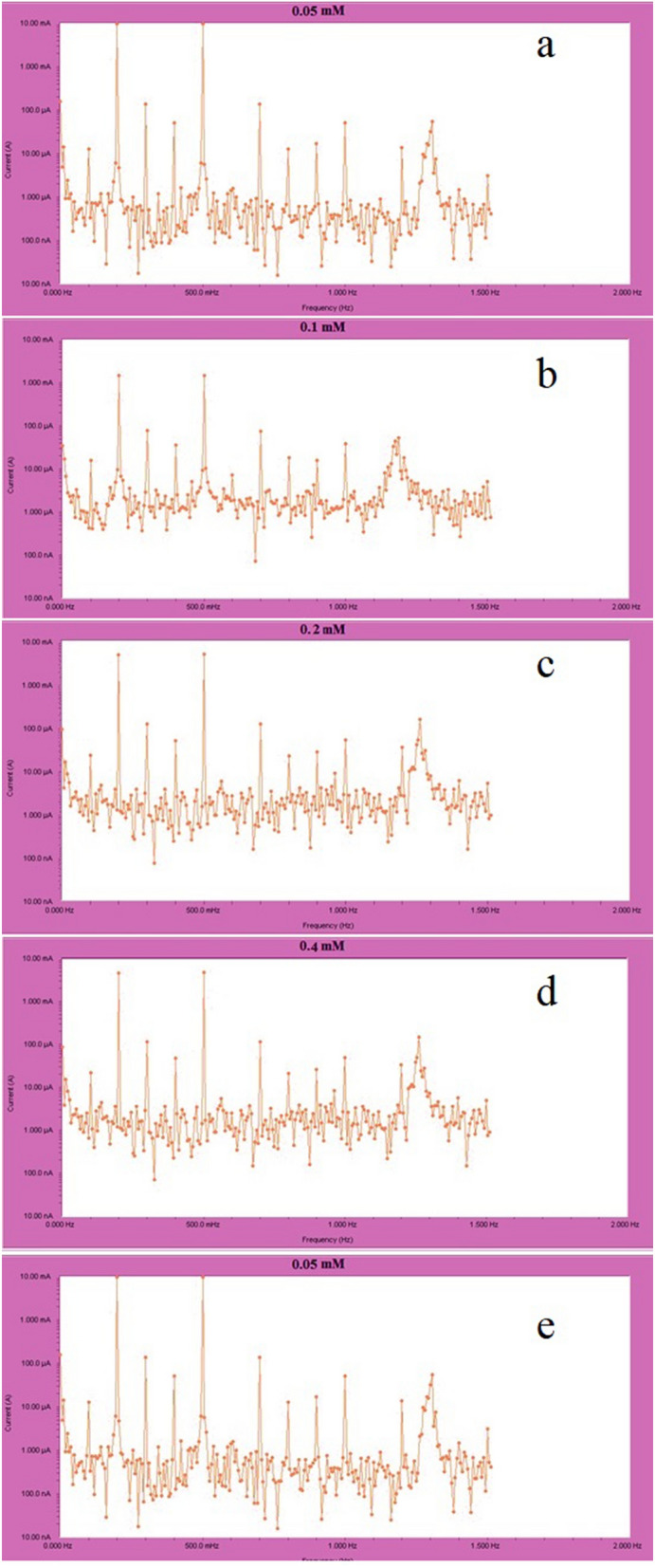
MS intermodulation spectrum in 1 M hydrochloric acid solution with (**a**) 0.05, (**b**) 0.1, (**c**) 0.2, (**d**) 0.4 and (**e**) 0.5 mM nonanedihydrazide at 303 K.

The current response spectrum as a function of frequency is the result of EFM experiments. Intermodulation spectra of electrochemical frequency modulation of MS in 1 M HCl in the absence and presence of different concentrations of corrosion inhibitor at 303 K are shown in Fig. 15a–e.

## DFT calculations

### Computational studies

The eigenvalues of the highest occupied (HOMO) and lowest unoccupied (LUMO) molecular orbitals, the HOMO–LUMO gap, electronegativity, chemical hardness, dipole moment, Fukui indices, and other parameters are among the most popular molecular-electronic properties in the inhibition efficiency correlation approach. The molecular-electronic properties in the inhibition efficiency correlation method are based on two assumptions. The first is that these chemical characteristics are crucial reactivity markers that can be used to forecast the direction of inhibitor adsorption bonding. Typically, the higher the eigenvalue of HOMO, the greater the molecular electron donation to the metal substrate, and the lower the eigenvalue of LUMO, the greater the electron back-donation from surface states to the molecule; however, high and low imply a small HOMO0-LUMO gap, because ELUMO is larger than EHOMO^64–66^. In evaluating inhibitor molecules, theoretical chemistry approaches are as useful as experimental procedures and the efficacy of inhibitor molecules can be calculated using different quantum parameters without the need for investigation. The subjects of atomic charge, border molecular orbitals, and energy gap can be used to classify computational factors commonly used in quantum chemical examinations of corrosion^67^. In addition, parameters such as molecular activity, chemical structures, and corrosion inhibitor capacity must be determined. The steric hindrance or how the metal solution interface handles the inhibitor can be revealed by the optimal chemical structures of the inhibitor molecules. Another approach is to show how the inhibitor behaves electrochemically in the presence of orbital energies and differences in orbital energies. Frontier Molecular Orbitals (Energy of HOMO, and Energy LUMO), the parameter of softness calculated from E_HOMO_, and the parameter of hardness calculated from E_LUMO_ are all carefully related to the inhibitor's potential to interact. The protection efficacy and levels of energy of the molecular orbitals of many organic compounds have been determined using computational chemistry research^68^. The Density Function Theory (DFT)^69^, which is based on the idea that a molecule's total electron energy is determined based on its electron density^70^, was used to investigate inhibitory behaviour in several different base sets of corrosion inhibitors. The electronic characteristics^71^ were computed and shown in Table 9 using Eqs. (4) to (9).

**Table 9:** Theoretical parameters calculated based on DFT (d,p) basis set at the B3LYP level of nonanedihydrazide.

Quantum characteristics	Nonanedihydrazide
${\text{E}}_{{{\text{HOMO}}}} { }\left( {{\text{eV}}} \right)$	−10.386
${\text{E}}_{{{\text{LUMO}}}} { }\left( {{\text{eV}}} \right)$	2.449
$\Delta {\text{E}} = {\text{E}}_{{{\text{HOMO}}}} - {\text{E}}_{{{\text{LUMO}}}} { }\left( {{\text{eV}}} \right)$	12.835
Dipole moment (μ) (D)	6.9978
Global hardness (η)	6.4175
Global softness (σ)	0.155
Electronegativity (χ)	3.9685

**Figure 16:**
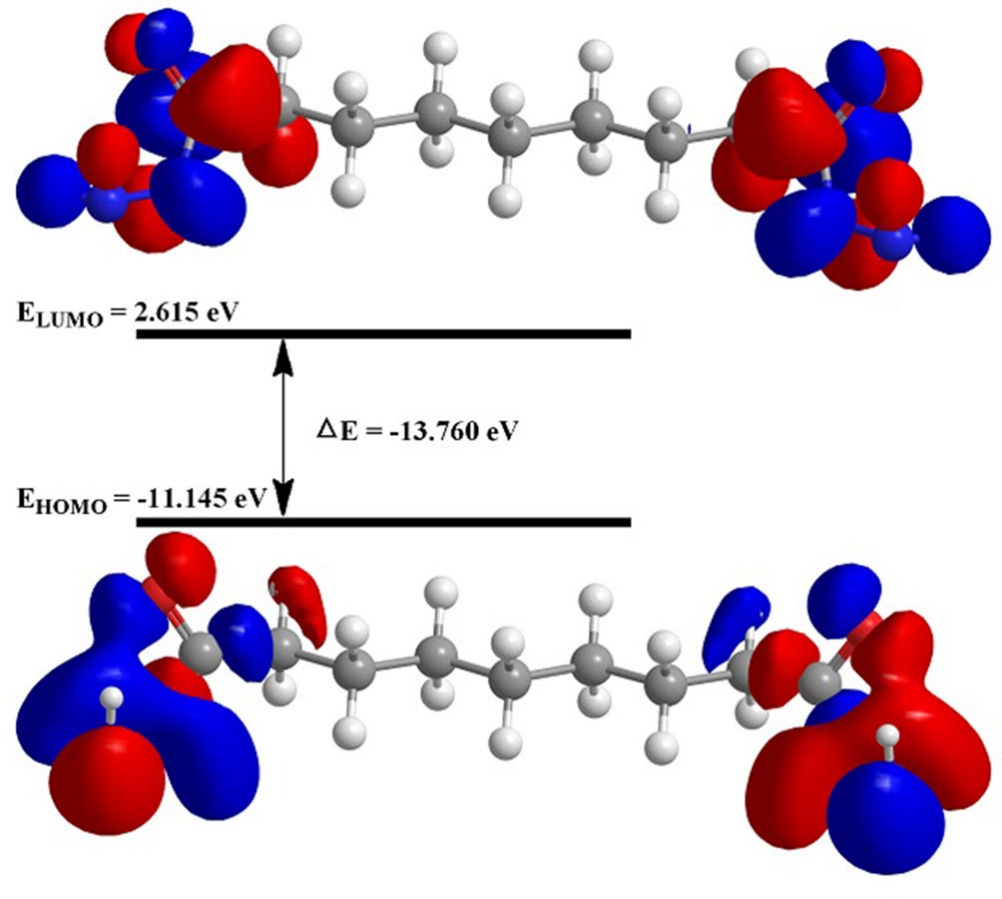
Inhibitor energy diagram HOMO and LUMO energies.

As EHOMO is linked to the electron-donating potential, increasing the value of HOMO increases the inhibitor's inhibition efficacy^72^, which is the mechanism for transferring the charge along the metal surface and initiating the adsorption mechanism. The assessed inhibitor is recognised as having the most significant inhibitory effectiveness depending on the greatest energy value of HOMO presented in Fig. 16 because it has a high value of inhibitory effectiveness.

The ability to receive electrons is fundamental to E_LUMO_, whereby a low-value E_LUMO_ indicates that the inhibitor molecules can find another negative charge on the MS surface. The LUMO values of the inhibitors studied were relatively high, with a high E_HOMO_ value. It was determined that the examined inhibitor molecules were reactive when acting as the donor, indicating that they are very efficient. Although inhibitor molecules with a small E_HOMO_ value reduce metal reactivity, the metal acts as a donor to the inhibitor molecules, thus, the inhibitor efficiency is lowered while the metal reactivity is boosted. The assessed inhibitor compounds had the greatest effective corrosion inhibition according to E_HOMO_-E_LUMO_ (Fig. 16). The inhibitor molecules with a high E_HOMO_ and a low energy gap were found to have high softness and low hardness values, indicating significant inhibitory activity. An additional component for inhibitory potency is electronegativity (χ) and electronegativity values determined for the tested inhibitor compounds may reveal information about the covalent bonds between the inhibitor and the metal surface^73^. The inhibitory effects of inhibitor molecules designed as an iron-inhibitor were studied (Table 9), indicating that the Fe atoms will form chemical bonds by gaining electrons from inhibitor molecules. The inhibitor is effective with a low electronegativity value based on the electronegativity value. The ΔN value in Table 9 shows that the examined inhibitor molecules transfer more electrons to the Fe atoms on the metal surface, indicating a higher performance. The dipole moment (μ) is another unique factor shown in Table 9. While previous studies have not found a significant correlation between dipole moment and corrosion inhibition efficiency, the inhibitory efficiency suggests that a high dipole moment signals strong corrosion inhibition potency. In different studies, it was found that lowering the dipole moment value increased corrosion inhibition efficiency. The assessed inhibitor has a low dipole moment value, suggesting a firmer coating of the metal surface when the dipole moment value is low.

### Mulliken charges

Table 10 shows the Mulliken atomic charges of the studied inhibitor. Mulliken charges is a widely used technique for predicting the interactions between adsorption sites^74^. By donating and receiving electrons, the heteroatoms in the inhibitor molecules boost the capacity to adsorb on the MS surface^75^. The inhibitor is efficient due to oxygen and nitrogen atoms in the inhibitor molecules and Table 10 also shows that the inhibitor molecules bonded coordinationaly with the d-orbital of Fe atoms on the MS surface via the oxygen and nitrogen atoms [O(10), O(13), N(11), N(12), N(14), and N(15)].

**Table 10:**
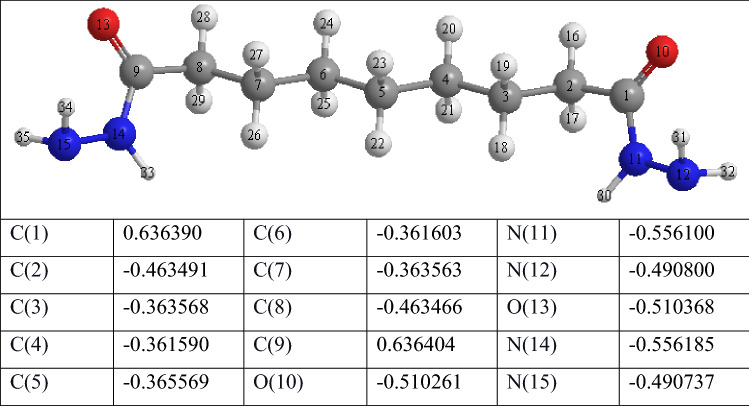
Calculated Mulliken charges of the inhibitor molecule atoms.

### Fukui functions

The Fukui functions were evaluated to verify the local interaction of the molecules. The Fukui function is defined as the first derivative of the system's electronic density (r) with respect to the number of electrons (N) at a constant external voltage v (r), as given by Eq. (16):

16 $$f(r) = \left( {\frac{\partial \rho (r)}{{\partial N}}} \right)_{{_{v(r)} }} = \left( {\frac{\partial \mu }{{\partial v(r)}}} \right)_{v(r)}$$

Some researchers in 1999 used the right and left derivatives about the number of electrons to determine the electrophilic and nucleophilic Fukui functions of the k site in a molecule (17–19):

17 $$f_{k}^{ + } (r) = \rho_{k} (N + 1) - \rho_{k} (N)\;{\text{for}}\;{\text{nucleophilic}}\;{\text{attack}}$$

18 $$f_{k}^{ - } (r) = \rho_{k} (N) - \rho_{k} (N - 1)\;{\text{for}}\;{\text{electrophilic}}\;{\text{attack}}$$

19 $$f_{k}^{0} (r) = \frac{{\rho_{k} (N + 1) - \rho_{k} (N - 1)}}{2}\;{\text{for}}\;{\text{radical}}\;{\text{attack}}$$

where ρk(N), ρk(N–1), and ρk(N + 1) are the gross electronic populations of the site k in the neutral, cationic, and anionic systems, respectively.

Recently, researchers proposed Δf (k), a dual descriptor defined as the difference between the nuclear and electrophilic Fukui functions (20):

20 $$\Delta f(k) = f_{k}^{ + } - f_{k}^{ - }$$

Similarly, Equation has been used to determine the corresponding dual local softness (21):

21 $$\Delta \sigma_{k} = \sigma_{k}^{ + } - \sigma_{k}^{ - } = \sigma \Delta f_{k}$$

### Local reactivity

**Figure 17:**
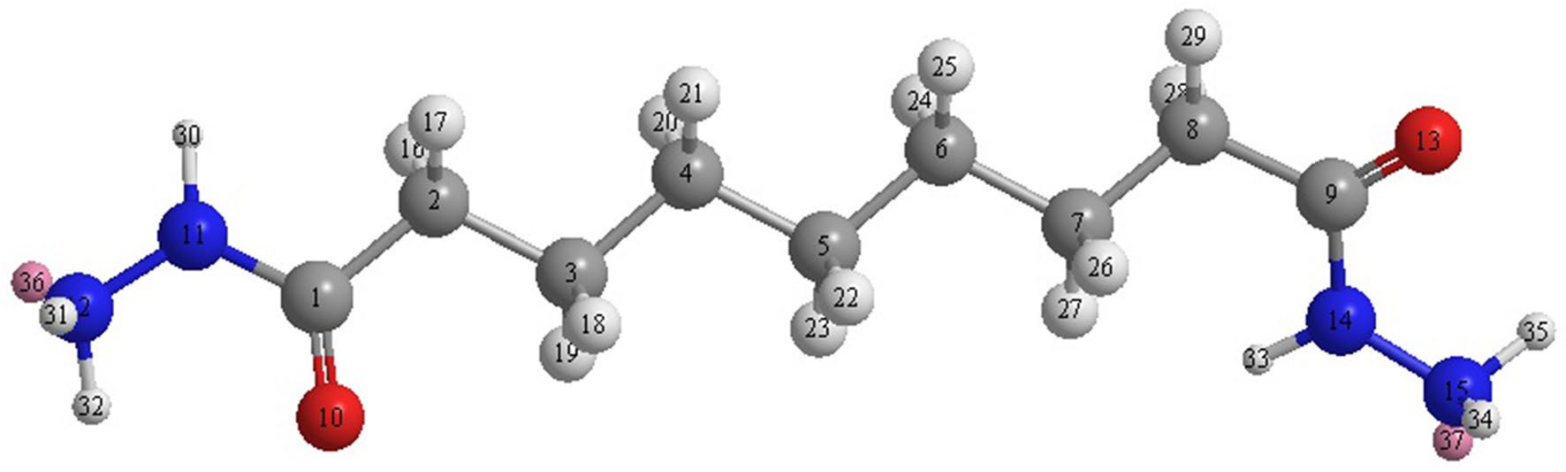
Fukui functions of the studied inhibitor.

The Fukui indices for each atom in the inhibitors were determined at the B3LYP/6–31++G level for a better understanding of the local reactivity of the tested inhibitor. The Fukui indices and local descriptors provide more complete information about the reactivity of the compounds under investigation and can help distinguish each portion of the inhibitor molecule based on its chemical activity with various substituent functional groups. As a result, the nucleophilic attack site will be where the value of f − is at its highest, whereas f + controls the site for electrophilic assault. The results show that the atoms O(10), O(13), N(11), N(12), N(14), and N(15) have the highest ${\text{f}}^{ - } {\text{kfk}}^{ - }$ values f-or nucleophilic attack, suggesting a proclivity to donate electrons to unoccupied molecular orbitals on the iron surface, resulting in the formation of a coordinate bond. This is consistent with the computed HOMO density. The atoms O(10), O(13), N(11), N(12), N(14), and N(15) in the investigated inhibitor had the greatest ${\text{f}}^{ - } {\text{kfk}}^{ - }$ values for the electrophilic attack, indicating that these are the locations most capable of accepting electrons to form feedback bonds with the Fe surface. This is also consistent with the LUMO orbital density calculated and supported by the values of the local dual indices (Δf, Δσ, and Δω), which show that this inhibitor has numerous active sites, with most centres having values of the two descriptors less than 0, except for a few atoms that have values greater than 0, indicating electrophilic centres. A closer look reveals that the carbon atoms have undergone back-donation, which corresponded to the border orbital results (Fig. 17). These findings suggest that the tested inhibitor molecule will have numerous active sites that will interact with the iron substrate, most likely locations with N and O atoms which are the most plausible sites for attaching to the iron surface via electron donation to the Fe 3d orbitals^76^. Furthermore, the metal surface and the tested inhibitor molecule may have a strong bond.

### Proposed protection mechanism

**Figure 18:**
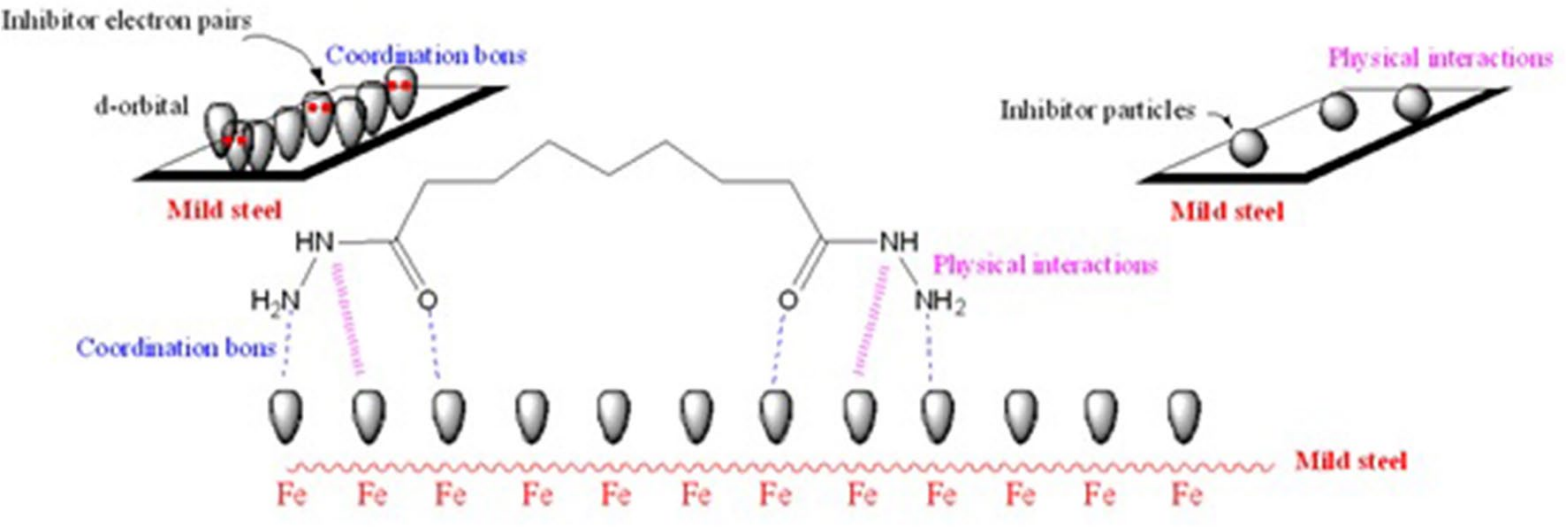
Proposed protection mechanism for MS by the inhibitor via chemical and physical adsorption processes.

The corrosion protection efficiency on the MS surface in an acidic environment can be exhibited based on the size of the inhibitor molecular chemical structure, the interaction modes with the iron atoms of the metal surface include the nature of the bonds (chemisorption) and the number of adsorption sites (physisorption). The N and O atoms in the inhibitor molecule all operate as adsorption sites, thus the inhibitor might use unpaired electrons to establish coordination bonds and chemisorb onto the MS surface. The protonation of nitrogen atoms is simple and can be accomplished through physisorption with chloride ions. The presence of unpaired electrons and the inductive impact of methylene groups are credited with the inhibitor's high inhibitive potency. On the steel/HCl interface, Fig. 18 depicts a possible blocked mechanism of the adsorption inhibitor molecules. Furthermore, the free electrons of the nitrogen and oxygen atoms are transferred to the d-orbitals of the iron atoms.

## Conclusion

The physical and chemical interactions between a metal and its environment cause changes in its characteristics, such as corrosion, which might alter the function, therefore corrosion inhibitors are often used to protect the metallic surface from corrosion. A corrosion inhibitor was synthesised and fully characterised using FTIR, ^1^H NMR, ^13^C NMR and mass spectrometry. The analysis revealed that:

1. Nonanedihydrazide is an effective corrosion inhibitor of MS in an HCl environment. The corrosion was controlled through the adsorption of inhibitor molecules onto the metal surface. As a mixed-type inhibitor, nonanedihydrazide showed outstanding inhibitory effectiveness, with increasing efficacy as the nonanedihydrazide concentration increased but decreased correspondingly as the temperature increased.
2. The inhibition efficiency obtained from mass loss measurements agreed with the EIS experimental results and polarisation techniques in which the treated environment had a higher value than the untreated one. As the concentration of nonanedihydrazide increased, the inhibitor inhibitive value increased.
3. The Langmuir isotherm model was used to explain the adsorption of nonanedihydrazide molecules on the MS surface.
4. The low E_LUMO_ of the nonanedihydrazide molecules combined with the high E_HOMO_ reveals that the nonanedihydrazide molecules were reactive by serving as a donor, hence confirming the predicted inhibition. The electronegativity atoms have a significant effect on the corrosion inhibition efficiency of nonanedihydrazide molecules, and the atom with a negative charge has a HOMO centre, so the nonanedihydrazide molecule coordinates with the iron atoms d-orbitals on the surface MS from these negatively charged atoms.
5. Nonanedihydrazide molecules show significant corrosion protection properties and are thought to be more effective when heteroatoms are added to the structure.

## References

[1] Kaczerewska O, Leiva-Garcia R, Akid R, Brycki B (2017). Efficiency of cationic gemini surfactants with 3-azamethylpentamethylene spacer as corrosion inhibitors for stainless steel in hydrochloric acid. J. Mol. Liq..

[2] Goyal M, Kumar S, Bahadur I, Verma C, Ebenso EE (2018). Organic corrosion inhibitors for industrial cleaning of ferrous and non-ferrous metals in acidic solutions: a review. J. Mol. Liq..

[3] Chauhan DS, Quraishi M, Sorour A, Saha SK, Banerjee P (2019). Triazole-modified chitosan: a biomacromolecule as a new environmentally benign corrosion inhibitor for carbon steel in a hydrochloric acid solution. RSC Adv..

[4] Sukul D, Pal A, Saha SK, Satpati S, Adhikari U, Banerjee P (2018). Newly synthesized quercetin derivatives as corrosion inhibitors for MS in 1 M HCl: combined experimental and theoretical investigation. Phys. Chem. Chem. Phys..

[5] Li, H. Qiang, Y. Zhao, W. Zhang, S. 2-Mercaptobenzimidazole-inbuilt metal-organic-frameworks modified graphene oxide towards intelligent and excellent anti-corrosion coating, Corrosion Science 191 (2021) 109715

[6] Qiang Y, Li H, Lan X (2020). Self-assembling anchored film basing on two tetrazole derivatives for application to protect copper in sulfuric acid environment. J. Mater. Sci. Technol..

[7] Elwan, H. A.; Zaky, M. T.; Farag, A. S.; Soliman, F. S.; Ezel Dean Hassan, M. A coupled extractive-oxidative process for desulfurization of gasoline and diesel fuels using a bifunctional ionic liquid. *J. Mol. Liq.* **2017**, *248*, 549– 555. doi:10.1016/j.molliq.2017.10.077

[8] Dutta A, Saha SK, Banerjee P, Patra AK, Sukul D (2016). Evaluating corrosion inhibition property of some Schiff bases for MS in 1 M HCl: competitive effect of the heteroatom and stereochemical conformation of the molecule. RSC Adv..

[9] Fouda AS, El-Askalany A, El-Habab A, Ahmed S (2019). Anticorrosion properties of some nonionic surfactants on carbon steel in 1 M HCl environment. J. Bio- Tribo-Corros..

[10] Shafek SH, Abubshait SA, Abubshait HA, Negm NA (2019). Antimicrobial potentials and surface activities of novel di-Schiff base nonionic surfactants bearing unsaturated hydrophobic tails. J. Mol. Liq..

[11] Berisha A, Podvorica F, Mehmeti V, Syla F, Vataj D (2015). Theoretical and experimental studies of the corrosion behavior of some thiazole derivatives toward MS in sulfuric acid media. Macedonian J. Chem. Chem. Eng..

[12] Mohsenifar F, Jafari H, Sayin K (2016). Investigation of thermodynamic parameters for steel corrosion in acidic solution in the presence of N, N′-Bis (phloroacetophenone)-1, 2 propanediamine. J. Bio Tribo Corros..

[13] Verma C, Quraishi M, Olasunkanmi L, Ebenso EE (2015). L-Proline-promoted synthesis of 2-amino-4-arylquinoline-3-carbonitriles as sustainable corrosion inhibitors for MS in 1 M HCl: experimental and computational studies. RSC Adv..

[14] Qianga Y, Zhangb S, Wang L (2019). Appl. Surf. Sci..

[15] Qiang, Y. Guo, L. Zhang, S. Li, W. Yu, S. Tan, J. Synergistic effect of tartaric acid with 2,6-diaminopyridine on the corrosion inhibition of mild steel in 0.5 M HCl. Scientific Reports 6 (2016) 33305. 33305; doi: 10.1038/srep33305 (2016). 10.1038/srep33305 PMC5024118 27628901

[16] Ashassi-Sorkhabi H, Majidi M, Seyyedi K (2004). Investigation of inhibition effect of some amino acids against steel corrosion in HCl solution. Appl. Surf. Sci..

[17] Migahed MA, El-Rabiei MM, Nady H, Elgendy A, Zaki EG, Abdou MI, Noamy ES (2017). Novel ionic liquid compound act as sweet corrosion inhibitors for X-65 carbon tubing steel: experimental and theoretical studies. J. Bio- Tribo-Corros..

[18] Odewunmi NA, Umoren SA, Gasem ZM (2015). Utilization of watermelon rind extract as a green corrosion inhibitor for mild steel in acidic media. J. Ind. Eng. Chem..

[19] Da Rocha J. C.; Gomes J. A. D. C. P.; D’Elia E. Corrosion inhibition of carbon steel in hydrochloric acid solution by fruit peel aqueous extracts. *Corros. Sci*. 2010, 52, 2341–2348. doi:10.1016/j.corsci.2010.03.033.

[20] Li L, Zhang X, Lei J, He J, Zhang S, Pan F (2012). Adsorption and corrosion inhibition of Osmanthus fragran leaves extract on carbon steel. Corros. Sci..

[21] El-Etre AY (2007). Inhibition of acid corrosion of carbon steel using aqueous extract of olive leaves. J. Colloid Interface Sci..

[22] ASTM G1. *Standard Practice for Preparing, Cleaning, and Evaluating Corrosion Test Specimens*; ASTM, 1999.

[23] Chauhan, D. S., Quraishi, M. A., Jafar Mazumder, M. A., Ali, S. A., Aljeaban, N. A., Alharbi, B. G. Design and synthesis of a novel corrosion inhibitor embedded with quaternary ammonium, amide and amine motifs for protection of carbon steel in 1 M HCl. *J. Mol. Liq.* **2020,** *317 *, 113917. 10.1016/j.molliq.2020.113917

[24] Onyeachu, I. B., Chauhan, D. S., Quraishi, M. A., Obot, I. B. Influence of hydrodynamic condition on 1,3,5-tris(4-methoxyphenyl)-1,3,5-triazinane as a novel corrosion inhibitor formulation for oil and gas industry. *Corros. Eng. Sci. Technol.* **2020,** *4 *, 1–8. 10.1080/1478422X.2020.1827348

[25] Dheeraj Singh Chauhan, M.A. Jafar Mazumder, M.A. Quraishi, K.R. Ansari, R.K. Suleiman. Microwave-assisted synthesis of a new Piperonal-Chitosan Schiff base as a bio-inspired corrosion inhibitor for oil-well acidizing. International Journal of Biological Macromolecules **2020,** *158 *, 231–243. 10.1016/j.ijbiomac.2020.04.195 10.1016/j.ijbiomac.2020.04.195 32344086

[26] Yıldız R (2015). An electrochemical and theoretical evaluation of 4, 6- diamino-2-pyrimidinethiol as a corrosion inhibitor for MS in HCl solutions. Corros. Sci..

[27] Pearson RG (1993). Chemical hardness: a historical introduction.

[28] John S, Joseph A, Sajini T, Jose AJ (2017). Corrosion inhibition properties of 1, 2, 4-hetrocyclic systems: electrochemical, theoretical and Monte Carlo simulation studies. Egypt. J. Pet..

[29] Koopmans T (1933). Ordering of wave functions and eigen-energies to the individual electrons of an atom. Physica.

[30] Plakhutin, B. N., Davidson, E. R. Koopmans' theorem in the restricted open-shell Hartree−Fock method. 1. A variational approach. *J. Phys. Chem. A. ***2009**, *113*(45):12386–12395. 10.1021/jp9002593 19459641

[31] Becke, A. D. Density-functional thermochemistry. IV. A new dynamical correlation functional and implications for exact-exchange mixing. *J. Chem. Phys*. 1996, 104(3), 1040–1046

[32] Fan, Y. X.; Han, Y. C.; Wang, Y. L. Effects of molecular structures on aggregation behavior of Gemini surfactants in aqueous solutions. *Acta Phys.–Chim. Sin*. 2016, 32, 214– 226. doi:10.3866/PKU.WHXB201511022

[33] Zhang Y, Pan Y, Li P, Zeng X, Guo B, Pan J, Hou L, Yin X (2021). Novel Schiff base-based cationic Gemini surfactants as corrosion inhibitors for Q235 carbon steel and printed circuit boards. Colloids Surf., A.

[34] Nahlé, A., Salim, R., El Hajjaji, F., Aouad, M. R., Messali, M., Ech-Chihbi, E., Hammouti, B., Taleb, M. Novel triazole derivatives as ecological corrosion inhibitors for mild steel in 1.0 M HCl: experimental & theoretical approach. *RSC Adv.* 2021;11(7):4147–62. 10.1039/d0ra09679b PMC8694344 35424362

[35] Espinoza-Vázquez A, Rodríguez-Gómez FJ, Martínez-Cruz IK, Ángeles-Beltrán D, Negrón-Silva GE, Palomar-Pardavé M, Romero LL, Pérez-Martínez D, Navarrete-López AM. Adsorption and corrosion inhibition behaviour of new theophylline–triazole-based derivatives for steel in acidic medium. *R. Soc. Open Sci.* 2019;6(3):181738. 10.1098/rsos.181738 PMC6458416 31032030

[36] Merimi I, Benkaddour R, Lgaz H, Rezki N, Messali M, Jeffali F, Oudda H, Hammouti B (2019). Insights into corrosion inhibition behavior of a triazole derivative for mild steel in hydrochloric acid solution. Materials Today: Proceedings..

[37] Wang L, Zhu MJ, Yang FC, Gao CW (2012). Study of a triazole derivative as corrosion inhibitor for mild steel in phosphoric acid solution. Int. J. Corros..

[38] Bentiss F, Bouanis M, Mernari B, Traisnel M, Vezin H, Lagrenee M (2007). Understanding the adsorption of 4H–1, 2, 4-triazole derivatives on mild steel surface in molar hydrochloric acid. Appl. Surf. Sci..

[39] El Mehdi B, Mernari B, Traisnel M, Bentiss F, Lagrenee M (2003). Synthesis and comparative study of the inhibitive effect of some new triazole derivatives towards corrosion of mild steel in hydrochloric acid solution. Mater. Chem. Phys..

[40] Hassan HH, Abdelghani E, Amin MA (2007). Inhibition of mild steel corrosion in hydrochloric acid solution by triazole derivatives: Part I. Polarization and EIS studies. Electrochimica Acta..

[41] Ramesh S, Rajeswari S (2004). Corrosion inhibition of mild steel in neutral aqueous solution by new triazole derivatives. Electrochim. Acta.

[42] Qiu LG, Xie AJ, Shen YH (2005). A novel triazole-based cationic gemini surfactant: synthesis and effect on corrosion inhibition of carbon steel in hydrochloric acid. Mater. Chem. Phys..

[43] Mert BD, Mert ME, Kardaş G, Yazıcı B (2011). Experimental and theoretical investigation of 3-amino-1, 2, 4-triazole-5-thiol as a corrosion inhibitor for carbon steel in HCl medium. Corros. Sci..

[44] Mazhar AA, Arab ST, Noor EA (2002). Influence of N-heterocyclic compounds on the corrosion of AlSi alloy in hydrochloric acidEffect of pH and temperature. Corrosion.

[45] Oguzie EE, Njoku VO, Enenebeaku CK, Akalezi CO, Obi C (2008). Effect of hexamethylpararosaniline chloride (crystal violet) on MS corrosion in acidic media. Corros. Sci..

[46] Wei Z, Duby P, Somasundaran P (2003). Pitting inhibition of stainless steel by surfactants: an electrochemical and surface chemical approach. J. Colloid Interface Sci..

[47] Negm, N. A.; Al Sabagh, A. M.; Migahed, M. A.; Abdel Bary, H. M.; El Din, H. M. Effectiveness of some diquaternary ammonium surfactants as corrosion inhibitors for carbon steel in 0.5 M HCl solution. Corros. Sci. 2010, 52, 2122.

[48] Ferreira ES, Giacomelli C, Giacomelli FC, Spinelli A (2004). Evaluation of the inhibitor effect of-ascorbic acid on the corrosion of MS. Mater. Chem. Phys..

[49] Oguzie EE, Enenebeaku CK, Akalezi CO, Okoro SC, Ayuk AA, Ejike EN (2010). Adsorption and corrosion-inhibiting effect of Dacryodis edulis extract on low-carbon-steel corrosion in acidic media. J. Colloid Interface Sci..

[50] Hsu CH, Mansfeld F (2001). Technical note: concerning the conversion of the constant phase element parameter Y 0 into a capacitance. Corrosion.

[51] Rammelt U, Reinhard G (1990). On the applicability of a constant phase element (CPE) to the estimation of roughness of solid metal electrodes. Electrochim. Acta.

[52] Dodson R, King L (1945). The reaction of Ketones with halogens and Thiourea. J. Am. Chem. Soc..

[53] Al-Amiery, A. A; Kadhum, A. A. H.; Mohamad, A. B.; Junaedi, S. *Materials*. 2013, 6(4):1420–1431. 10.3390/ma6041420 PMC5452315 28809218

[54] Junaedi, S.; Al-Amiery, A. A.; Kadihum, A.; Kadhum, A. A.; Mohamad, A. B. *Int J Mol Sci.* **2013**, *14*(6):11915–11928. 10.3390/ijms140611915 PMC3709763 23736696

[55] Kadhum, A. A. H.; Mohamad, A. B.; Hammed, L. A.; Al-Amiery, A. A.; San, N. H.; Musa, A. Y. *Materials*, **2014**, *7*(6):4335–4348. 10.3390/ma7064335 PMC5455929 28788680

[56] Al-Amiery AA, Kadhum AAH, Mohamad AB, Musa AY, Li CJ. *Materials*. **2013**, *6*(12), 5466-5477 10.3390/ma6125466 PMC5452761 28788402

[57] Cao C (1996). On electrochemical techniques for interface inhibitor research. Corros. Sci..

[58] Obot IB, Obi-Egbedi NO (2010). Theoretical study of benzimidazole and its derivatives and their potential activity as corrosion inhibitors. Corros. Sci..

[59] Deng S, Li X, Xie X (2014). Hydroxymethyl urea and 1,3-bis(hydroxymethyl) urea as corrosion inhibitors for steel in HCl solution. Corros. Sci..

[60] Qu Q, Jiang S, Bai W, Li L (2007). Effect of ethylenediamine tetraacetic acid disodium on the corrosion of cold rolled steel in the presence of benzotriazole in hydrochloric acid. Electrochim. Acta.

[61] Haque J, Srivastava V, Chauhan DS, Lgaz H, Quraishi MA (2018). Microwave-induced synthesis of chitosan schiff bases and their application as novel and green corrosion inhibitors: experimental and theoretical approach. ACS Omega.

[62] Hsu CH, Mansfeld F (2001). Technical note: concerning the conversion of the constant phase element parameter Y_0_ into a capacitance. Corrosion.

[63] Feliu S (2020). Electrochemical impedance spectroscopy for the measurement of the corrosion rate of magnesium alloys: brief review and challenges. Metals.

[64] Kokalj, A., Lozinšek, M. Kapun, B. Taheri, P. Neupane, S. Losada-Pérez, P. Xie, S. Stavber, S. Crespo, D. Renner, F. Mol, A. Milošev, I. Simplistic correlations between molecular electronic properties and inhibition efficiencies: do they really exist?. Corrosion Science, V. 179, 2021, 108856.

[65] Qiang Y, Guo L, Li H, Lan X (2021). Fabrication of environmentally friendly Losartan potassium film for corrosion inhibition of mild steel in HCl medium. Chem. Eng. J..

[66] Veneranda M, Aramendia J, Bellot-Gurlet L, Colomban P, Castro K, Madariaga J (2018). FTIR spectroscopic semi-quantification of iron phases: a new method to evaluate the protection ability index (PAI) of archaeological artefacts corrosion systems. Corros. Sci..

[67] Singh AK, Chugh B, Singh M, Thakur S, Pani B, Guo L, Kaya S, Serdaroglu G (2021). Hydroxy phenyl hydrazides and their role as corrosion impeding agent: a detail experimental and theoretical study. J. Mol. Liq..

[68] Chugh B, Singh AK, Chaouiki A, Salghi R, Thakur S, Pani B (2020). A comprehensive study about anti-corrosion behavior of pyrazine carbohydrazide: gravimetric, electrochemical, surface and theoretical study. J. Mol. Liq..

[69] Ichchou I, Larabi L, Rouabhi H, Harek Y, Fellah A (2019). Electrochemical evaluation and DFT calculations of aromatic sulfonohydrazides as corrosion inhibitors for XC38 carbon steel in acidic media. J. Mol. Struct..

[70] Obot, I. B.; Macdonald, D. D.; Gasem, Z. M. Density functional theory (DFT) as a powerful tool for designing new organic corrosion inhibitors. Part 1: An overview. *Corros. Sci.* **2015**, *99*, 1– 30, DOI: 10.1016/j.corsci.2015.01.037

[71] El Ibrahimi, B.; Jmiai, A.; El Mouaden, K.; Oukhrib, R.; Soumoue, A.; El Issami, S.; Bazzi, L. Theoretical evaluation of some α-amino acids for corrosion inhibition of copper in acidic medium: DFT calculations, Monte Carlo simulations and QSPR studies. *J. King Saud Univ., Sci.* 2018, in press, DOI: 10.1016/j.jksus.2018.04.004

[72] Priya Kumari Paul, Mahendra Yadav. Investigation on corrosion inhibition and adsorption mechanism of triazine-thiourea derivatives at MS / HCl solution interface: Electrochemical, XPS, DFT and Monte Carlo simulation approach. *J. Electroanal. Chem.* **2020,** *877 *, 114599. 10.1016/j.jelechem.2020.114599

[73] Ahamad; I., Prasad; R., Quraishi; M. A. Adsorption and inhibitive properties of some new Mannich bases of isatin derivatives on corrosion of MS in acidic media. *Corros. Sci*. 2010, 52, 1472.

[74] Fang; J., Li; J. Quantum chemistry study on the relationship between molecular structure and corrosion inhibition efficiency of amides. *J. Mol. Struct*. 2002, 593, 179.

[75] Uwaya GE, Fayemi OE, Sherif EM, Junaedi H, Ebenso EE (2020). Synthesis, electrochemical studies, and antimicrobial properties of Fe_3_O_4_ nanoparticles from callistemon viminalis plant extracts. Materials.

[76] Yang W, Mortier WJ (1986). The use of global and local molecular parameters for the analysis of the gas-phase basicity of amines. J. Am. Chem. Soc..

